# Trends in hypertension prevalence, control, and antihypertensive use in England from 2003 to 2021: insights from annual, nationwide Health Surveys for England

**DOI:** 10.1136/bmjmed-2025-001556

**Published:** 2025-11-27

**Authors:** Catherine Graham, James Steckelmacher, Jai Prashar, Ayesha Ahmed, Maxim Capel, Neil R Poulter, Peter Sedgwick Sever, Ajay Kumar Gupta

**Affiliations:** 1 William Harvey Research Institute, Clinical Pharmacology and Precision Medicine, Queen Mary University of London, London, UK; 2 Barts Health NHS Trust, London, UK; 3 London, UK; 4 Imperial Clinical Trials Unit, School of Public Health, Imperial College London, London, UK; 5 National Heart and Lung Institute, Imperial College London, London, UK; 6 NIHR Barts Biomedical Research Centre, Barts Health NHS Trust and Queen Mary University of London, London, UK

**Keywords:** Hypertension, Public health, Epidemiology, Health policy, Primary health care

## Abstract

**Objective:**

To determine the annual trends over two decades in hypertension diagnosis, true hypertension prevalence, and control and use of antihypertensive drugs in England.

**Design:**

Annual, representative, nationwide Health Surveys for England from 2003 to 2021.

**Setting:**

Nationally representative sample consisting of participants from private households, identified through stratified, national, multistage sampling.

**Participants:**

67 242 people older than 16 years surveyed between 2003 and 2021.

**Results:**

Between 2003 and 2021, the prevalence of measured hypertension decreased significantly from 37.8% in 2003 to 33.2% in 2018 (average annual percentage change (AAPC) −0.9%, 95% confidence interval −1.6% to −0.6%). Mean population systolic and diastolic blood pressure decreased significantly between 2003 to 2019 (systolic blood pressure from 128.7 to 124.0 mm Hg, AAPC −0.15%, −0.21% to −0.11%; diastolic blood pressure from 73.7 to 71.8 mm Hg, AAPC −0.12%, −0.20% to −0.04%). The prevalence of those with undiagnosed hypertension in the community decreased from 32.6% in 2003 to 23.7% in 2011, gradually worsening thereafter, with a steep increase in 2021 when 32.4% of those with hypertension were undiagnosed (AAPC −1.04%, −1.92% to −0.22%). Among people diagnosed (known) with hypertension, the proportion of those with blood pressure controlled to target increased across the study period (AAPC 1.66%, 0.04% to 2.27%), but this was mainly driven by the significant increase from 47.3% in 2003 to 56.5% in 2009 (AAPC 3.62%, 1.52% to 5.74%). A plateau was observed from 2011 onwards when there was no further significant improvement in the proportion of people with diagnosed (known) hypertension and blood pressure controlled to target. People with diagnosed (known) but uncontrolled hypertension showed a small but significant decrease in the mean number (standard deviation) of antihypertensive drugs used during the study period from 2.17 (0.86) in 2003 to 2.14 (0.96) in 2021 (AAPC −0.18%, −0.68% to −0.03%). Those with diagnosed (known) and controlled hypertension had no significant change in the mean number of drugs used.

**Conclusions:**

These findings indicate that there was a significant improvement in hypertension prevalence and control in the 2000s, with a plateau after 2011. The prevalence of undiagnosed hypertension in the community reduced in the early 2000s and 2010s, but has since returned to the same level as two decades ago.

**WHAT IS ALREADY KNOWN ON THIS TOPIC**

- Hypertension is a major cause of cardiovascular mortality in England
- Between 1994 and 2011 in England, mean blood pressure in the general population improved
- Across this period, awareness, treatment, and control rates for people with hypertension improved significantly

**WHAT THIS STUDY ADDS**

- This study shows that despite a significant improvement in hypertension prevalence and control in the 2000s, it plateaued after 2011, and possibly worsened after the covid-19 pandemic
- Hypertension prevalence after the covid-19 pandemic is similar to two decades ago
- An association between this rise in hypertension and an upsurge in premature cardiovascular mortality was observed

**HOW THIS STUDY MIGHT AFFECT RESEARCH, PRACTICE, OR POLICY**

- Rising hypertension prevalence is likely contributing to the increase in premature cardiovascular deaths observed in recent years
- More data are required for the years since the covid-19 pandemic to establish any trends in hypertension in England after 2021 and assess whether the pandemic had a lasting impact on hypertension prevalence, awareness, and control

## Introduction

 From 1990 to 2019, the number of people with hypertension worldwide more than doubled, meaning that hypertension now affects one in three adults.^1^ Globally, there is evidence that increasing numbers of people have hypertension that is undiagnosed or uncontrolled. This is of great concern because hypertension is the leading preventable risk factor for cardiovascular disease, stroke, and all cause mortality, and is responsible for one in five deaths globally.^2 3^ However, according to the World Health Organization (WHO), 76 million deaths, 120 million strokes, 79 million heart attacks, and 17 million heart failure events could be averted by 2050 if even half of those with high blood pressure had this brought under control.^1^ However, heterogeneity exists among countries across the income and development spectrum in prevalence, detection, and control of hypertension, with some middle income countries outperforming many high income nations.^1^

The cost saving benefits of treating hypertension with drugs are well documented.^4^ Additionally, implementing comprehensive preventative, early detection, and effective management programmes for hypertension over the next three decades could be significant, with WHO estimating a cost saving of $18 (£13.50; €15) for every $1 spent.^1^ Yet in England, the estimated annual cost of hypertension to the NHS is more than £2bn, and cardiovascular disease as a whole costs the wider economy in England an estimated £15.8bn each year.^5 6^

In England, the annual, nationally representative Health Surveys for England (HSE) have been providing information on population health since 1991. Between 1994 and 2011 in England, mean blood pressure for men and women in the general population and in the population of those with a hypertension diagnosis improved, with those treated for hypertension seeing an improvement in mean blood pressure from 150/80.4 mm Hg to 135.4/73.5 mm Hg.^5^,^7^ Based on the strong positive linear association from 1994 to 2011 in the proportion of people with treated and controlled hypertension, in 2014, it was suggested that 80% of patients treated for hypertension would have satisfactorily controlled blood pressure levels by 2022—a change predicted to potentially have prevented between 35 000 and 55 000 fatal cardiovascular and other mortality events compared with 2011.^7^ This projection is in stark contrast to a recently reported upsurge in cardiovascular mortality.^8^

This study aims to build on previous work by Falaschetti and colleagues^7^ by providing a detailed analysis of trends in hypertension in England from 2003 to 2011 and beyond. To date, we are unaware of any studies examining hypertension trends since the marked improvements of the 2000s (characterised by seminal studies^9^ and changes to hypertension treatment algorithms^10^ in the 2000s and 2010s). Additionally, studies are required that include English hypertension prevalence data since the start of the covid-19 pandemic to investigate whether the subsequent disturbance to society, healthcare contact, and the provision and delivery of healthcare services affected blood pressure trends in England.

In summary, we aimed to evaluate annual, nationwide, representative survey data, with directly measured blood pressure, to determine whether the improvement in blood pressure prevalence, diagnosis, and control before 2011 in England was sustained thereafter. We also aimed to assess treatment trends, in particular the class and number of common antihypertensive drugs prescribed to people with hypertension in England, and to evaluate the impact of the covid-19 pandemic on blood pressure trends in England.

## Methods

Data for this study come from 14 cross sectional, nationally representative surveys from the HSE series between 2003 and 2021 (specifically 2003, 2006, and 2009-21 inclusive). Details of the survey methods are described elsewhere.^11^ In summary, each annual HSE used multistage, stratified, random probability sampling to measure health and health related behaviours of the non-institutionalised population in England, with new participants recruited every year. Data collection involved an interview followed by a nurse visit, both in the participant's home. During the interview, information about sociodemographic data, risk factors, and medical history was collected. At the nurse visit, blood pressure measurements and use of prescribed drugs were recorded, and non-fasting blood samples were taken. English mid-year population estimates and premature cardiovascular mortality statistics for 2003-22 were derived from Office for National Statistics data.

Each participant gave verbal consent to be interviewed, visited by a nurse, and have blood pressure and anthropometric measurements taken, and also gave written consent for blood sampling. Sociodemographic information collected by the interviewers included self-assigned ethnic group. Participants who had eaten, drunk alcohol, or smoked in the 30 minutes before blood pressure measurements were excluded from analyses according to the HSE protocols.

Hypertension was defined as doctor diagnosed hypertension or the use of antihypertensive drugs, or mean systolic blood pressure or mean diastolic blood pressure measurement greater than 140 or 90 mm Hg, respectively (the threshold for diagnosis from national guidelines since 1999; online supplemental table 1). Antihypertensive drugs were defined as angiotensin converting enzyme inhibitors, beta blockers, calcium channel blockers, diuretics, or other blood pressure lowering drugs (specifically vasodilators, centrally acting antihypertensives, sympatholytics, and alpha blockers). The proportion of people with diagnosed hypertension was defined (according to guidelines available at the time of the survey) as those who self-reported they had hypertension diagnosed by a doctor or those who were taking a prescribed antihypertensive drug. To estimate the proportion of people with hypertension and controlled blood pressure, control to two target thresholds was included: control to the blood pressure target advised by guidelines available at the time of that particular survey; and retrospective control to the most common blood pressure target in current English guidelines (≤140/90 mm Hg). For the surveys we analysed, other than the year 2003 when the target clinic blood pressure was ≤140/85 mm Hg, control and diagnostic thresholds in all subsequent years correspond to a target blood pressure ≤140/90 mm Hg. These definitions were then used to categorise the population into the following groups:

- Diagnosed (known) hypertension and controlled blood pressure
- Diagnosed (known) hypertension and uncontrolled blood pressure
- Raised blood pressure (undiagnosed hypertension)—present on blood pressure measurements taken by a nurse on the day of evaluation (ie, those in the community who were unaware and therefore undiagnosed).

Blood pressure was measured by using Omron HEM207 monitors, with three readings taken from each participant in a seated position at one minute intervals using an appropriately sized cuff after a five minute rest. Data used in this study were based on the mean of the second and third measurements. In our analysis and based on the available ethnic group data across the study period, we recoded these self-assigned ethnicities into four categories: white, black/black British/Caribbean/African, Asian, and other (mixed or multiple ethnic groups).

### Statistical analysis

We limited analyses to participants who were 16 years and older with valid blood pressure measurements. We excluded those who had missing data for doctor diagnosed hypertension and whether they were prescribed antihypertensive drugs to allow us to determine whether participants belonged to the diagnosed (known) hypertension category.

Of 17 potential surveys, we included those with complete data, allowing us to categorise people as having diagnosed or undiagnosed and controlled or uncontrolled hypertension. The 2004 HSE survey was excluded because it only collected blood pressure from a subset of people, and the 2007 and 2008 HSE surveys were excluded because they did contain information about doctor diagnosed hypertension.

Survey weights were produced by HSE from 2003 onwards to minimise bias owing to non-response or oversampling at crucial stages of the interviews (interviewer visit, nurse visit, blood sampling). All relevant weights were applied for our analyses from 2003 onwards. Transformations were applied to the data when required, as advised by HSE, to mitigate for changes in survey equipment during the two decades.

Joinpoint regression models (using a weighted least squares estimator) were used to summarise trends and significant changes in prevalence or control over the study period. Joinpoints were identified using a permutation test and Monte Carlo sampling; the most parsimonious model was selected in an automated process using the weighted Bayesian information criterion.^12^ Changes in a variable over time were summarised using the average annual percentage change (AAPC).

Data analyses were conducted using Python (version 3.8.10). All statistical coding was reviewed by at least two members of the team. Joinpoint Regression Programme (version 5.2.0.0) from the National Cancer Institute was used to identify changes in trends in hypertension prevalence, proportion diagnosed (known), systolic and diastolic blood pressure, and proportion of the population with controlled blood pressure, and to assess the statistical significance of trends. We assessed the prevalence of each antihypertensive category, overall and in those younger than 55 years, and 55 years or older. We generated 95% confidence intervals (CI) and P values to identify significant trends.

**Figure 1:**
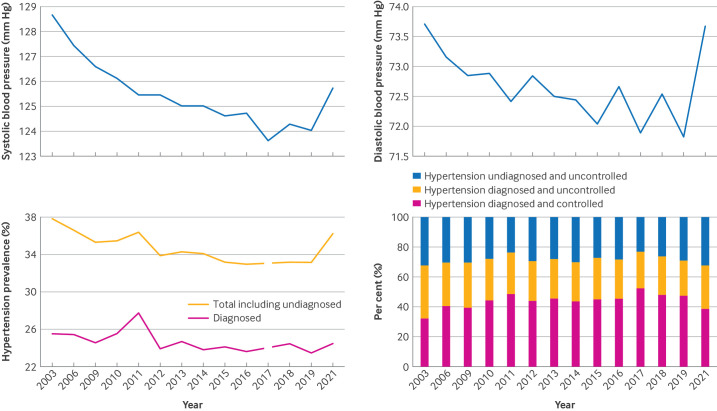
Trends in systolic blood pressure, diastolic blood pressure, prevalence of hypertension, and blood pressure control among people diagnosed with hypertension and those undiagnosed in England from 2003 to 2021. Top left: mean systolic blood pressure; top right: mean diastolic blood pressure; bottom left: population prevalence of hypertension—total and diagnosed; bottom right: hypertension control prevalence (of all people with hypertension) according to definitions of diagnosis and control during that period

**Figure 2:**
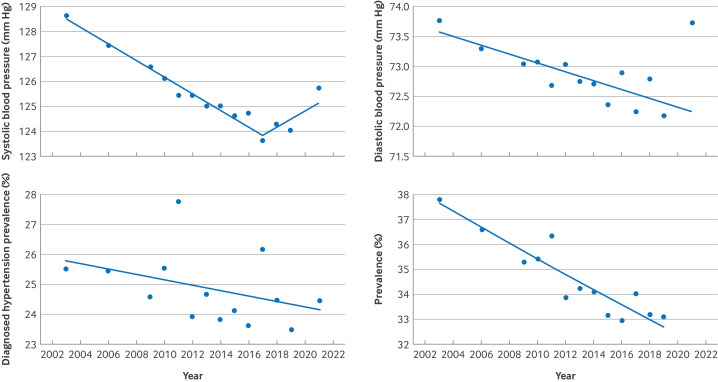
Joinpoint regression models evaluating annual trends in systolic blood pressure, diastolic blood pressure, prevalence of diagnosed hypertension, and total prevalence of hypertension (including undiagnosed) in England from 2003 to 2021. Top left: mean systolic blood pressure (AAPC 2003-17 −0.26%, 95% confidence interval −0.32% to −0.23%, P<0.001; AAPC 2017-21 0.23%, −0.11% to 0.60%, P=0.200). Top right: mean diastolic blood pressure. Bottom left: prevalence of diagnosed hypertension. Bottom right: prevalence of hypertension (AAPC 2003-18 −0.93%, −1.61% to −0.57%, P=0.011; AAPC 2018-21 2.51%, −1.54% to 4.88%, P=0.417). AAPC=average annual percentage change

**Figure 3:**
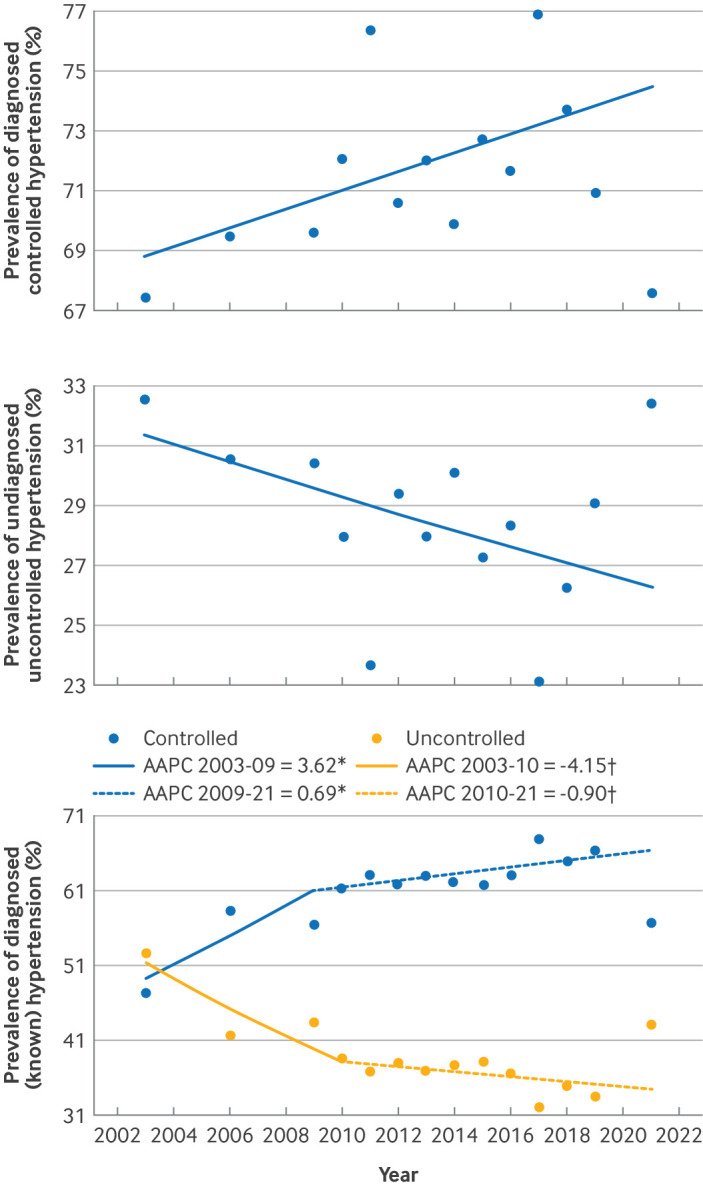
Joinpoint regression models evaluating annual trends among people with hypertension, showing proportions with diagnosed (known) and undiagnosed hypertension, and proportions with blood pressure control among people with diagnosed hypertension in England from 2003 to 2021. Top: prevalence of diagnosed (known) hypertension; middle: prevalence of undiagnosed, uncontrolled hypertension; bottom: prevalence of diagnosed (known) and controlled and diagnosed (known) and uncontrolled hypertension. *Controlled: AAPC 2003-09 3.62%, 95% confidence interval 1.52% to 5.74%, P<0.001; AAPC 2009-21 0.69%, −7.95% to 1.39%, P=0.591. †Uncontrolled: AAPC 2003-10 −4.15%, −5.99% to −2.43%, P<0.001; AAPC 2010-21 −0.90%, −2.50% to 4.88%, P=0.635. AAPC=average annual percentage change

**Figure 4:**
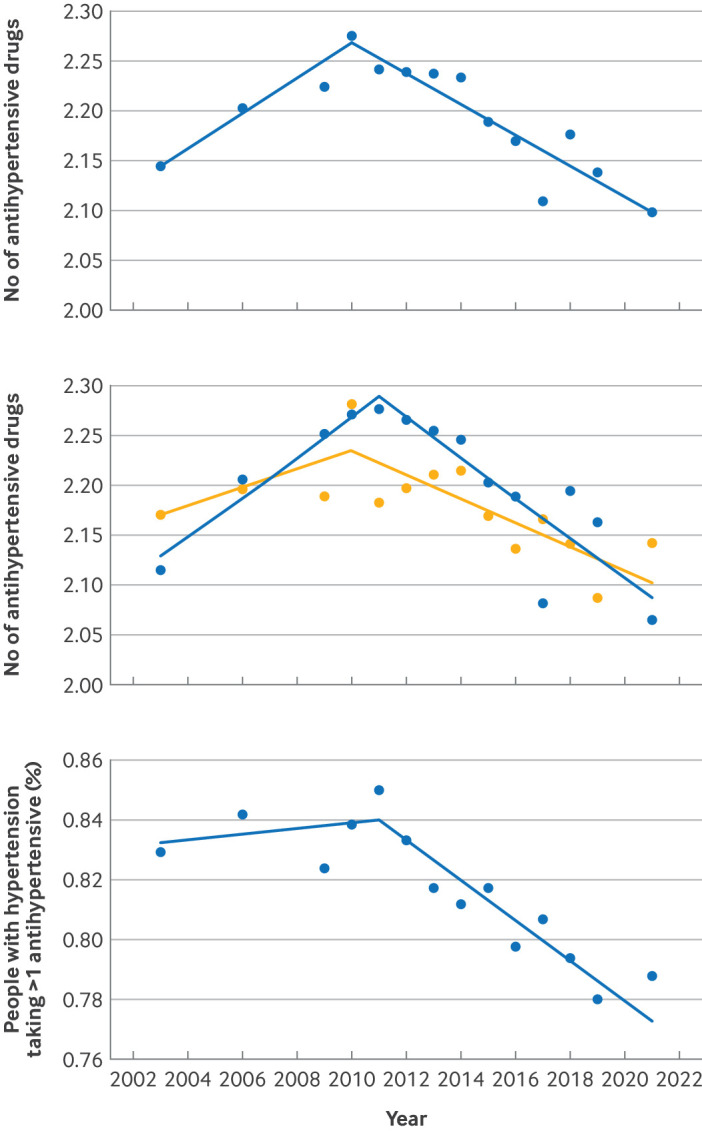
Joinpoint regression models evaluating annual trends in mean number of antihypertensive agents used among people with diagnosed (treated) hypertension, with controlled and uncontrolled blood pressure, and prevalence of use of more than one antihypertensive agent. Top: mean number of antihypertensives used among people with diagnosed (treated) hypertension (AAPC 2003-10 0.80%, 95% confidence interval 0.40% to 1.40%, P=0.001; AAPC 2010-21 −0.71%, −1.06% to −0.52%, P<0.001). Middle: mean number of antihypertensives used among people with diagnosed (treated) but uncontrolled (orange), and diagnosed (treated) controlled hypertension (blue) (controlled: AAPC 2003-11 0.91%, 0.37% to 2.04%, P=0.002; AAPC 2011-21 −0.91%, −1.70% to −0.56%, P<0.001; uncontrolled: AAPC 2003-10 0.43%, −0.06% to 1.07%, P=0.111; AAPC 2010-21 −0.55%, −3.33% to −0.33%, P=0.003). Bottom: prevalence of use of more than one hypertensive agent among people with diagnosed (treated) hypertension (AAPC 2003-11 0.13%, −0.27% to 0.88%, P=0.492; AAPC 2011-21 −0.86%, −4.00% to −0.63%, P<0.001). AAPC=average annual percentage change

Because of the limited number of data points before 2003, specifically only HSE 1994 and HSE 1998 collecting relevant information on hypertension, the ability to discern reliable trends before 2003 was constrained. As a result, trends reported in this paper focused on 2003-21 only. Trends across the period from 1994 to 2021 can be found in online supplemental table 2 and figures 1-5. The study was conducted in accordance with the Strengthening the Reporting of Observational Studies in Epidemiology (STROBE) checklist (online supplemental file 2).

### Patient and public involvement

Patients and the public were not involved in producing this retrospective study.

## Results

In surveys between 2003 and 2021, 121 822 adults aged 16 or older were interviewed and 67 334 adults aged 16 or older had valid systolic and diastolic blood pressure measurements. A further 92 people were excluded because of missing data for doctor diagnosed hypertension and antihypertensive drug use. After the exclusions, 67 242 participants were included in the analysis (online supplemental table 3).

The mean weighted age of the sample was 49.0 years, and 51.9% were female; 89.7% were white, 2.4% were black, black British, Caribbean or African, 5.1% were Asian, and 2.6% were of other or mixed ethnicity, according to self-reported data. Online supplemental table 3 shows the demographic characteristics of the population by year, including age, sex, and self-reported ethnicity.

Mean systolic blood pressure fell over the period (AAPC −0.15%, 95% CI −0.21% to −0.11%, P<0.001) with a consistent fall from 128.7 mm Hg in 2003 to 124.0 mm Hg in 2019 (AAPC 2003-17 −0.26%, −0.23% to −0.32%, P<0.001) and little evidence of an increase in systolic blood pressure rising thereafter to 125.7 mm Hg by 2021 (AAPC 2017-21 0.23%, −0.11% to 0.60%, P=0.200; figure1  figure 2**,** online supplemental figure 6 and table 4). Mean diastolic blood pressure reduced from 73.7 mm Hg in 2003 to 71.8 mm Hg in 2019, with a subsequent rise to 73.7 mm Hg by 2021 (AAPC −0.12%, −0.20% to −0.04%, P=0.004; figure 1, online supplemental figure 7 and table 4)

The population prevalence of total hypertension (diagnosed (known) and undiagnosed) fell over the study period from 37.8% in 2003 and to 36.2% in 2021 (AAPC −0.37%, −1.06% to −0.02%, P=0.04) with little evidence of an increase from 2018 to 2021 (AAPC 2018-21 2.51%, −1.54% to 4.88%, P=0.417; figures1  2**,** online supplemental table 4).

The population prevalence of doctor diagnosed hypertension reduced minimally from 25.5% in 2003 to 24.4% in 2021, with no significant trend detected through Joinpoint regression models (AAPC −0.37%, −0.92% to 0.11%, P=0.109; figures1  2, online supplemental table 4).

Over the study period, we saw a significant decrease in the proportion of people in the community found to have hypertension, but whose hypertension was undiagnosed, for whom a reduction was seen from 32.6% in 2003 to 23.7% in 2011; although this proportion had risen to 32.4% by 2021 (AAPC −1.04%, −1.92% to −0.22%, P=0.012; figure 3**,** online supplemental table 4).

Of those identified with diagnosed (known) hypertension according to the study definition (see methods), the proportion with blood pressure control increased across the overall study period (AAPC 1.66%, −0.04% to 2.27%, P<0.001; figure 3**,** online supplemental table 4). However, this was driven mainly by the sharp and significant rise in this proportion from 47.3% in 2003 to 56.5% in 2009 (AAPC 2003-09 3.62%, 1.52% to 5.74%, P<0.001) and then 63.1% in 2011, with no further significant change until 2021, when it dropped to 56.8% (AAPC 2009-21 0.69%, −7.95% to 1.39%, P=0.591; figure 3). Inversely, the proportion of people with hypertension diagnosed and treated, but whose blood pressure was uncontrolled to target showed a significant decrease from 52.7% in 2003 to 33.5% in 2019, followed by a non-statistically significant increase to 43.23% in 2021 (AAPC −2.18%, −2.96% to −1.22%, P=0.046; figure 3**,** online supplemental table 4).

The mean number (standard deviation) of antihypertensive drugs used by people with diagnosed (known) hypertension did not change significantly during the study period from 2.14 (0.85) in 2003 to 2.10 (0.92) in 2021 (AAPC −0.12%, −0.28% to 0.01%, P=0.061; figure 4). Within this group, there was a small but significant decrease in the mean number of antihypertensive drugs used by people with diagnosed (known) hypertension whose blood pressure was uncontrolled from 2.17 (0.86) in 2003 to 2.14 (0.96) in 2021 (AAPC −0.18%, −0.68% to −0.03%, P=0.026), but not in those with controlled hypertension (AAPC −0.11%, −0.42% to 0.15%, P=0.286; figure 4, online supplemental table 5).

From 2003 to 2011, between 82% and 85% of people with diagnosed (treated) hypertension were prescribed more than one antihypertensive, falling to about 79% by 2021 (AAPC −0.42%, 95% CI −1.00% to −0.24%, P<0.001; figure 4, online supplemental table 5). The prevalence of angiotensin converting enzyme inhibitor use in people with diagnosed (treated) hypertension increased significantly over the study period, with a sharp increase from 26.7% in 2003 to 46.1% in 2010, followed by a period of stasis from 2011 to 2021 on the background of an overall increasing trend (AAPC 3.60%, 2.80% to 4.41%, P<0.001; online supplemental table 6 and figure 8). Calcium channel blocker use in people with diagnosed hypertension fell from 81.0% in 2003 to 67.3% in 2021 (AAPC −1.27%, −1.57% to −1.02%, P<0.001), and a similar significant decline in diuretic use was also observed across the study period (online supplemental table 6 and figure 8). The prevalence of beta blocker use in those diagnosed with hypertension initially fell from 24.2% in 2003 to 18.8% in 2011, with no significant change noted between 2011 and 2021 and no overall trend across the study period (AAPC −0.46%, −1.90% to 0.54%, P=0.361; online supplemental table 6 and figure 8). Trends in drug use in those diagnosed with hypertension stratified by age (<55, ≥55) showed similar trends (online supplemental table 7), with a significant increase in the proportion of people younger than 55 using angiotensin converting enzyme inhibitors, and a statistically significant reduction in the use of calcium channel blockers among the same group.

## Discussion

### Principal findings

This longitudinal study of hypertension prevalence and control in England had several key findings. Despite a significant improvement in prevalence, diagnosis, and control of hypertension in England in the 2000s, a clear stagnation from the 2010s onwards was seen in many key measures of diagnosis and control. Only 38.3% of people with hypertension have achieved adequate control; previous projections^7^ estimated that if improvements in all aspects of management seen from 1994 to 2011 continued at the same rate, 80% of the treated population would have controlled blood pressure by 2022—by this measure, only minimal progress has been made since 2011. According to this analysis, undiagnosed hypertension rates remain high, with around one third of people with hypertension remaining undiagnosed, and therefore untreated.

This study shows a significant and pronounced reversal of progress between 2019 and 2021, with hypertension prevalence, control, and diagnosis rates almost returning to the same levels as the early to mid 2000s. These trends, alongside decreasing use of several antihypertensive drugs and in the context of worsening population level control, may represent increasing physician and health service inertia. Finally, we show that these results correlate closely with a newly increasing trend in premature cardiovascular mortality, highlighting the potential consequence of worsening hypertension control at the population level.

### Study implications

Our analyses show a marked deterioration in hypertension prevention, control, and diagnosis between 2019 and 2021. It is unclear from these data whether this decline can be attributed entirely to the covid-19 pandemic, or in part to a rapid reversal in trend owing to several factors. However, severe disruption to diagnosis and management efforts during the covid-19 pandemic due to social distancing and lockdown measures is likely.^13^ Disruption to primary care services probably affected access to routine blood pressure testing and contributed to lower detection rates, with reduced dispensing of antihypertensive drugs over the pandemic period possibly also contributing.^14^

National data on premature cardiovascular mortality rates show a surge in recent years, with premature cardiovascular mortality closely following hypertension prevalence for most of the study period (online supplemental figure 9). This finding accords with other data suggesting that hypertension is the most important modifiable factor in cardiovascular disease, and strongly underscores the importance of hypertension control in reducing avoidable cardiovascular mortality at the national level.

Our analysis shows that, as of 2021, hypertension prevalence remains high, affecting one in three adults. Additionally, hypertension often remains undiagnosed or uncontrolled. Despite marked improvements in prevalence and control from 1994 to 2011, it was followed by a notable plateau in progress from 2011 to 2019, and a major decline in progress by 2021. The driver of improvements in hypertension detection, management, and control before 2011, likely multifactorial, continues to be debated. For example, the Quality and Outcomes Framework that was introduced in 2004 as a pay-per-performance scheme aimed to incentivise general practitioners to deliver effective interventions for long term conditions such as hypertension.^15^ However, renumeration was based on the liberal target of achieving a blood pressure of less than 150/90 mm Hg, so it is unlikely that this framework can be credited for all the progress made during the 2000s. Other factors related to overall healthcare investment, dietary, lifestyle, and socioeconomic trends and changes in the modifiable risk factors associated with developing hypertension are important in explaining how hypertension prevalence and control improved and then plateaued over these decades.

Reducing dietary salt intake, even modestly, significantly reduces blood pressure with a dose dependent effect.^16^ After the Food Standards Agency was established in 2000 and collaborated with organisations such as Action on Salt, and the Committee on Medical Aspects of Food and Nutrition Policy, a salt reduction strategy took effect from 2003 to 2011 that, among other initiatives, set national targets for salt intake of less than 6 g/day and worked with food industries to incrementally reduce levels of salt in their products. This strategy saw the salt content of food products reduced by 20-40%, with a concomitant reduction in population salt intake by 19% from 9.38 to 7.58 g/day between 2003 and 2014.^17^ Salt intake has since increased (to 8.39 g/day in 2018), which as Action on Salt notes, has coincided with the responsibility for salt reduction being handed over to the food industry and stalling in the setting of new salt targets. Action is clearly required to reverse this trend, and the reinvigoration of an independent salt reduction programme is a vital first step.^18^

Obesity prevalence has also increased over the study period. As of 2018, 63% of adults in England were classified as overweight or obese, with more than one in four adults in England classified as obese^19^; 78% and 65% of the risk for developing essential hypertension in men and women, respectively, is attributed to excess body mass,^20^ and is likely a substantial driver of the plateau in hypertension prevalence and control seen since 2011. It will be important to assess the longer term impact of the introduction of health policies that have come into force since 2018, such as the soft drinks industry levy or “sugar tax,”^21^ and the calorie labelling regulations,^22^ plus what therapeutic advances such as GLP-1 (glucagon-like peptide 1) agonists will have on obesity prevalence going forward.

Excessive alcohol consumption and a sedentary lifestyle are also known to contribute to the risk of hypertension.^23 24^ Data on alcohol consumption in adults in England also come from the HSE and generally show that, since 2012, the proportion of men and women drinking more than the weekly recommended limit of 14 units of alcohol has declined. The Active Lives Surveys have shown that about 22.6% of adults are physically inactive, doing less than 30 minutes of physical activity per week.^25^ Mental health disorders such as depression and psychological stress are also associated with an increased risk of developing hypertension^26 27^ and have increased in prevalence over the past two decades.^28^ Socioeconomic and neighbourhood deprivation is known to be associated with an increased risk of hypertension.^29^ As described in the 2020 report “Health equity in England: The Marmot review 10 years on,” in the context of “widespread and deep cuts in most areas of public spending” since 2010, many markers of economic inequality have increased, with higher rates of in-work poverty, increased food insecurity, stagnant wages, and reduced welfare benefits.^30^

This study offers insight into the use of antihypertensives over the past two decades (online supplemental tables 5-7 and figure 8). The mean number of antihypertensive drugs being prescribed to patients with treated but uncontrolled hypertension has fallen significantly over the period, and the proportion of those taking more than one antihypertensive drug has fallen over the period, which could suggest increasing therapeutic inertia^31^ as observed in other studies.^32^ Additionally, an increasing proportion of those with diagnosed (known) hypertension taking angiotensin converting enzyme inhibitors over the period may reflect increasing multimorbidity in people with hypertension, with these drugs more frequently prescribed for other common indications such as heart failure, diabetes, or chronic kidney disease.^33^ Furthermore, a reduction in the proportion of those taking calcium channel blockers (though most patients with diagnosed (known) hypertension, 65%, continue to take one), which could again reflect preferential selection of angiotensin converting enzyme inhibitors as first line agents in those with relevant comorbidities. Concurrently, a decreasing proportion of patients with diagnosed hypertension taking diuretics and beta blockers likely reflects the influence of landmark clinical trials^9^ feeding through to contemporary guidelines that prioritise renin-angiotensin system inhibitors and calcium channel blockers as first line agents of choice for hypertension over beta blockers and diuretics.^34^

### Strengths and limitations

This study had several strengths. It included a large, nationally representative sample of over 65 000 participants, undertaken with comparable questions year on year, so direct comparisons can be made over a 16 year period. The application of survey weights and transformations has been used to minimise bias from non-response and oversampling or changes in survey equipment during the two decades, respectively. Additionally, robust analytical methods (Joinpoint regression) were used to investigate the significance of periodic trends in blood pressure, control, and antihypertensive use across and throughout the study period. Finally, our study examines the impact of the pandemic period on blood pressure trends in England.

The study also had several limitations. Our classification of undiagnosed hypertension and uncontrolled blood pressure was based on the mean of two blood pressure measurements taken by the visiting nurse in the home, which could have overestimated uncontrolled and undiagnosed hypertension owing to a white coat effect. The use of home blood pressure monitoring and ambulatory blood pressure monitoring would usually mitigate such errors, but would not have been possible in the single visit HSE protocol. Additionally, our analysis stopped in 2021, before the end of the covid-19 pandemic; further, the presence of government restrictions and social distancing measures made it challenging to draw definite conclusions on the longer term impact of the pandemic based on these data alone. HSE did not take place in 2020 because of the covid-19 pandemic, and full datasets from the 2022 survey were not available to researchers at the time of writing. The effects of the covid-19 pandemic on blood pressure and hypertension trends should be assessed when these data are available, given the likelihood of longlasting effects on cardiovascular disease prevalence and control caused by the pandemic.^14^ At present, our data are insufficient to make a definitive claim of a post-pandemic worsening trend. It remains to be seen whether observed changes represent a temporary disruption or a continuation of the pre-pandemic trend of sustained erosion in previously achieved gains. The 2021 survey data used were limited in that they had a smaller sample size, reducing the study's power to detect pandemic related effects, and they differed in methodology (eg, virtual interviews were conducted), limiting the extent to which results can be compared with previous surveys. Finally, in this observational study, we were unable to definitively ascribe changes in, for example, blood pressure control, to changes in practice, improved availability, or efficacy of pharmacotherapy or other factors.

### Conclusions

In summary, our analysis of a large nationally representative sample of more than 65 000 adults in England has revealed that most improvements in blood pressure diagnosis and control were achieved before 2011 and plateaued over the 2010s, with notable rises in undiagnosed and uncontrolled hypertension at the onset of the covid-19 pandemic. These trends might be contributing to the excess cardiovascular disease deaths observed in recent years. Our data suggest that over five million people in England may be living with undiagnosed hypertension, and a further 4.9 million with diagnosed and uncontrolled hypertension. The reasons for this worsening trend will be multifactorial, but are likely a composite of changing health policy focus, lifestyle, dietary, and socioeconomic trends, with increasing physician focus and therapeutic inertia further contributing. To make up lost ground from the past decade, a multi-agency approach must be reinvigorated that engages all stakeholders in improving awareness, identification, and management of hypertension and its causes. Evidence indicates that prioritising enhanced diagnostic capabilities, minimising barriers to drug adherence, and emphasising education for healthcare providers and patients, alongside targeted health policy reforms, can substantially improve hypertension management and subsequently reduce premature cardiovascular mortality.

## Supplementary material

10.1136/bmjmed-2025-001556 Supplementary file 1

10.1136/bmjmed-2025-001556 Supplementary file 2

## Data Availability

Data are available in a public, open access repository.
