# Supplementary material for: Trends in hypertension prevalence, control, and antihypertensive use in England from 2003 to 2021: insights from annual, nationwide Health Surveys for England
Source: BMJ Med. 2025 Nov 27;4(1):e001556. doi: 10.1136/bmjmed-2025-001556 (PMC12666190; doi:10.1136/bmjmed-2025-001556)

Supplementary Material

Trends in hypertension prevalence, control and antihypertensive use in England over the last 2 decades: insights from annual, nationwide Health Surveys for England from 2003 to 2021

Contents

Supplementary Methods.

Supplementary Table 1: Summary of hypertension guidelines for diagnosis and control from 1999-2021 in England

Supplementary Table 2: Proportion of individuals in England with controlled *vs* uncontrolled hypertension in diagnosed and undiagnosed groups with ‘period’ vs ‘current’ definitions of diagnosis and control, 1994-2021.

Supplementary Table 3: Baseline Characteristics of Included Participants from 2003–2021.

Supplementary Table 4: Hypertension prevalence, proportion diagnosed, Mean SBP and DBP and BP control from 2003–2021.

Supplementary Table 5: Use of antihypertensives among those diagnosed with hypertension, 2003-2021.

Supplementary Table 6: Prevalence of individual antihypertensive medication use in those diagnosed with hypertension, 2003-2021.

Supplementary Table 7: Antihypertensive use by age (<55 or >55) and drug category, 2003-2021.

Supplementary Figure 1: Hypertension prevalence in England according to ’period’ and ’current’ definitions, 1994-2021.

Supplementary Figure 2: Hypertension control in England, according to ’period’ definitions of diagnosis and control, 1994-2021.

Supplementary Figure 3: Hypertension control in England, according to ‘current’ definitions of diagnosis and control, 1994-2021.

Supplementary Figure 4: Mean SBP (mmHg) in England, 1994-2021.

Supplementary Figure 5: Mean DBP (mmHg) in England, 1994-2021.

Supplementary Figure 6: Mean SBP (mmHg) amongst those with and without hypertension, according to ‘period’ definition, 2003-2021

Supplementary Figure 7: Mean DBP (mmHg) in England amongst those with and without hypertension according to ‘period’ definition, 2003-2021.

Supplementary Figure 8: Prevalence of ACE inhibitor, calcium channel blocker, beta-blocker and diuretic use in diagnosed hypertensives, 2003-2021.

Supplementary Figure 9: Overall cardiovascular risk factor prevalence and cardiovascular mortality in England, 2003-2022.

Supplementary Methods

For HSE 2021, data were collected from January 2021 to June 2022. As this transected the COVID-19 pandemic, interviews were administered via video and telephone calls for the 2021 survey.

The results presented in *Supplementary Table 2* and *Supplementary Figures 1-3* include data from 1994 to 2003. These should be interpreted in the context of a significant reduction in the diagnostic threshold for hypertension adopted from 1999 onwards, when the diagnostic threshold changed from BP > 160/95mmHg to BP > 140/90mmHg. Separate analyses against 'period' and 'current' diagnostic thresholds in *Supplementary Figures 1-3* are provided to account for this change.

For the 2015-2019 surveys, age was recorded as 5-year bands rather than an integer, and participants' ages were instead recoded to the median age within each age band to enable comparability.

Supplementary Table 1: Summary of hypertension guidelines for diagnosis and control from 1999-2019 in England.

**Year**	**Organisation**	**Hypertension Diagnosis**		**BP control target on treatment**	
		**Clinic BP (mmHg)**	**HBPM/ABPM (mmHg)**	**General (mmHg)**	**Diabetes (mmHg)**
1999	BHS	140/90	Not routinely recommended	Optimal clinic 140/85, ABPM 130/80.<br>Audit standard clinic 150/90, ABPM 140/85	Optimal clinic BP 140/80, ABPM 130/75.<br>Audit standard clinic 140/85, ABPM 140/80
2004	BHS	140/90	HBPM 135/85, ABPM 125/80	≤ 140/85	≤ 130/80
2006	NICE/BHS	140/90		≤ 140/90	≤ 130/80
2011	NICE/BHS	140/90	135/85	<80 years old:<br>≤ 140/90<br>> 80 years old:<br>≤ 150/90	NICE CG87: <140/80mmHg (below 130/80mmHg if there is kidney, eye or cerebrovascular disease)
2019	NICE	140/90	135/85	<80 years old:<br>≤140/90<br>>80 years old:<br>≤150/90	<80 years old:<br>T2DM: ≤140/90<br>T1DM with ACR <70mg/mmol: ≤140/90<br>T1DM with ACR >70mg/mmol: ≤ 130/80<br>>80 years old:<br>T1DM (regardless of ACR): ≤150/90

Supplementary Table 2: Proportion of individuals in England with controlled *vs* uncontrolled hypertension in diagnosed and undiagnosed groups with 'period' vs 'current' definitions of diagnosis and control, 1994-2021.

Measure	Year															
	1994	1998	2003	2006	2009	2010	2011	2012	2013	2014	2015	2016	2017	2018	2019	2021
Proportion of those with hypertension diagnosed and controlled (period) (%)	62.86	65.84	31.90	40.51	39.31	44.11	48.18	42.68	45.34	43.45	44.92	45.25	52.19	47.90	47.14	38.37
Proportion of those with hypertension diagnosed and controlled (current) (%)	38.01	38.85	35.12	40.51	39.31	44.11	48.18	42.68	45.34	43.45	44.92	45.25	52.19	47.90	47.14	38.37
Proportion of those with hypertension diagnosed and uncontrolled (period) (%)	20.58	20.58	35.55	28.96	30.26	27.92	28.17	26.90	26.67	26.43	27.80	26.41	24.68	25.82	23.80	29.22
Proportion of those with hypertension diagnosed and uncontrolled (current) (%)	45.43	47.57	32.51	28.96	30.26	27.92	28.17	26.90	26.67	26.43	27.80	26.41	24.68	25.82	23.80	29.22
Proportion of those with hypertension undiagnosed	16.57	13.58	32.55	30.53	30.43	27.97	23.65	29.42	27.99	30.12	27.28	28.34	23.13	26.28	29.07	32.42

and uncontrolled (period) (%)																
Proportion of those with hypertension undiagnosed and uncontrolled (current) (%)	16.57	13.58	32.55	30.53	30.43	27.97	23.65	29.42	27.99	30.12	27.28	28.34	23.13	26.28	29.07	32.42

Supplementary Table 3: Baseline Characteristics of Included Participants in the Health Surveys of England from 2003–2021.

Characteristic	Year													
	**2003**	**2006**	**2009**	**2010**	**2011**	**2012**	**2013**	**2014**	**2015**	**2016**	**2017**	**2018**	**2019**	**2021**
**No. of adults, unweighted (n)**	9086	8680	2777	4652	4753	4514	5133	4668	4523	4346	4387	4066	4164	1493
**Mean [SD] age (years)**	47.7 [18.2]	48.0 [18.6]	47.9 [18.6]	48.3 [18.7]	48.2 [18.7]	49.0 [18.8]	49.1 [18.8]	49.1 [18.9]	49.2 [19.0]	50.4 [18.8]	50.2 [18.8]	50.5 [18.7]	50.1 [18.9]	53.5 [18.4]
**Age group (n/%)**														
16-39	3288 (36.7)	2987 (35.2)	992 (35.4)	1573 (34.6)	1614 (35.0)	1449 (33.2)	1698 (33.7)	1531 (33.6)	1545 (34.8)	1301 (31.1)	1358 (32.2)	1228 (31.4)	1340 (33.2)	352 (26.5)
40-69	4375 (48.8)	4236 (49.9)	1390 (49.6)	2275 (50.1)	2295 (49.8)	2212 (50.7)	2529 (50.3)	2267 (49.8)	2155 (48.5)	2132 (51.0)	2064 (49.0)	1954 (50.0)	1949 (48.2)	651 (49.0)
≥70	1423 (14.5)	1463 (14.9)	395 (14.9)	803 (15.3)	844 (15.1)	853 (16.1)	906 (16.0)	870 (16.6)	823 (16.7)	913 (17.9)	965 (18.7)	884 (18.6)	875 (18.6)	325 (24.5)
**Sex (n/%)**														
Female	4620 (51.5)	4385 (51.7)	1462 (52.2)	2347 (51.7)	2411 (52.3)	2308 (52.9)	2602 (51.7)	2358 (51.8)	4441 (50.9)	2188 (52.3)	2214 (52.6)	2025 (51.8)	2109 (52.2)	702 (52.9)
**Ethnicity (n/%)**														
White	8382 (93.5)	7833 (92.3)	2536 (90.6)	4087 (90.0)	4142 (90.0)	3917 (89.7)	4471 (88.8)	4056 (89.1)	3923 (88.4)	3663 (87.6)	3694 (87.7)	3377 (86.4)	3493 (86.4)	1157 (87.2)
Black*	152 (1.7)	180 (2.1)	81 (2.9)	118 (2.6)	100 (2.2)	121 (2.8)	126 (2.5)	105 (2.3)	130 (2.9)	131 (3.1)	107 (2.5)	107 (2.7)	88 (2.2)	36 (2.7)
Asian	284 (3.2)	325 (3.8)	94 (3.3)	163 (3.6)	187 (4.1)	200 (4.6)	232 (4.6)	218 (4.8)	280 (6.3)	293 (7.0)	318 (7.6)	324 (8.3)	351 (8.7)	101 (7.6)
Other**	144 (1.6)	147 (1.7)	88 (3.1)	167 (3.7)	169 (3.7)	122 (2.8)	202 (4.0)	172 (3.8)	105 (2.4)	90 (2.2)	87 (2.1)	99 (2.5)	108 (2.7)	30 (2.3)

*Black, Black British, Caribbean or African. **Mixed or multiple ethnic groups. SD = Standard Deviation.

Supplementary Table 4: Hypertension prevalence, proportion of those previously diagnosed (known), mean SBP and DBP and BP control in known hypertensives in England, 2003–2021.

Measure	Year														Inflection points in trend (from Join-point model)	% change per year (+ means increase, - means decrease) AAPC (95% CI)	*p*-value for change in trend
	**2003**	**2006**	**2009**	**2010**	**2011**	**2012**	**2013**	**2014**	**2015**	**2016**	**2017**	**2018**	**2019**	**2021**			
No. of adults, unweighted (n)	9086	8680	2777	4651	4753	4514	5133	4668	4523	4346	4387	4066	4164	1493			
No. of adults, weighted sum (n)	9006	8569	2820	4603	4644	4375	5054	4570	4415	4163	4262	3907	4072	1462			
Prevalence of hypertension (%)	37.81	36.60	35.31	35.43	36.36	33.87	34.24	34.08	33.15	32.95	34.02	33.18	33.09	36.17	2018	-0.37 (-1.06 to -0.02)	0.042
Prevalence of diagnosed hypertension (%)	25.50	25.43	24.57	25.52	27.76	23.90	24.66	23.82	24.11	23.61	26.16	24.46	23.47	24.44	None	-0.37 (-0.92 to 0.11)	0.109
Proportion of hypertensive individuals with hypertension diagnosed (%)	67.45	69.47	69.57	72.03	76.35	70.58	72.01	69.88	72.72	71.66	76.87	73.72	70.94	67.58	None	+0.46 (0.05 to 0.82)	0.024

Mean SBP (mmHg) [SD]	128.7 [18.7]	127.4 [17.5]	126.6 [17.0]	126.1 [17.0]	125.5 [16.5]	125.5 [16.5]	125.0 [16.6]	125.0 [16.9]	124.6 [16.3]	124.7 [16.4]	123.6 [15.9]	124.3 [16.0]	124.0 [16.2]	125.7 [17.2]	2017	-0.15 (-0.22 to -0.11)	<0.001
Mean DBP (mmHg) [SD]	73.7 [11.5]	73.2 [10.9]	72.9 [10.9]	72.9 [11.1]	72.4 [10.8]	72.8 [10.9]	72.5 [10.9]	72.5 [11.2]	72.0 [10.9]	72.7 [10.9]	71.9 [10.8]	72.6 [10.7]	71.8 [10.8]	73.7 [10.8]	None	-0.12 (-0.20 to -0.04)	0.004
Population prevalence of individuals with hypertension diagnosed without BP control (%)	52.71	41.69	43.50	38.76	36.89	38.11	37.03	37.83	38.23	36.85	32.11	35.02	33.54	43.23	2010	-2.18 (-2.96 to -1.22)	0.046
Population prevalence of individuals with hypertension diagnosed with BP control (%)	47.29	58.31	56.50	61.24	63.11	61.89	62.97	62.17	61.77	63.15	67.89	64.98	66.46	56.77	2009	+1.66 (0.04 to 2.27)	<0.001
Population prevalence of individuals with undiagnosed hypertension (%)	32.55	30.53	30.43	27.97	23.65	29.42	27.99	30.12	27.28	28.34	23.13	26.28	29.07	32.42	None	-1.04 (-1.92 to -0.22)	0.012

Control is to ‘period definition’ (<u><</u>140/85 for 2003. <u><</u>140/90 for 2006-2021) SD = standard deviation; AAPC = average annual percentage change, CI = confidence interval. Weighted sum is shown using default sample weightings.

Supplementary Table 5: Use of antihypertensives among those diagnosed with hypertension, 2003-2021.

**Measure**	**Year**														Join-points	% change per year (+ means increase, - means decrease) AAPC (95% CI)	*p*-value for trend
	**2003**	**2006**	**2009**	**2010**	**2011**	**2012**	**2013**	**2014**	**2015**	**2016**	**2017**	**2018**	**2019**	**2021**			
Number of individuals with diagnosed hypertension (%)	25.50	25.43	24.57	25.52	27.76	23.90	24.66	23.82	24.11	23.61	26.16	24.46	23.47	24.44	None	-0.37 (-0.92 to 0.11)	0.109
Number of individuals on more than one antihypertensive medication (%)	83.0	84.2	82.4	83.9	85.0	83.3	81.8	81.2	81.8	79.8	80.7	79.4	78.0	78.8	2011	-0.42 (-1.00 to -0.24)	<0.001
Mean [SD] number of antihypertensives in individuals diagnosed with hypertension	2.14 [0.85]	2.2 [0.87]	2.22 [0.93]	2.27 [0.93]	2.24 [0.89]	2.24 [0.93]	2.24 [0.94]	2.23 [0.96]	2.19 [0.91]	2.17 [0.94]	2.11 [0.90]	2.18 [0.95]	2.14 [0.94]	2.10 [0.92]	2010	-0.12 (-0.28 to 0.01)	0.061
Mean [SD] number of antihypertensives in individuals with diagnosed and controlled hypertension	2.11 [0.83]	2.21 [0.85]	2.25 [0.90]	2.27 [0.92]	2.28 [0.89]	2.27 [0.95]	2.25 [0.96]	2.24 [0.97]	2.20 [0.91]	2.19 [0.94]	2.08 [0.89]	2.19 [0.96]	2.16 [0.95]	2.06 [0.89]	2011	-0.11 (-0.42 to 0.15)	0.286
Mean [SD] number of antihypertensives in individuals with diagnosed and uncontrolled hypertension	2.17 [0.86]	2.2 [0.90]	2.19 [0.96]	2.28 [0.93]	2.18 [0.90]	2.20 [0.89]	2.21 [0.93]	2.21 [0.93]	2.17 [0.90]	2.14 [0.93]	2.17 [0.91]	2.14 [0.95]	2.09 [0.93]	2.14 [0.96]	2010	-0.18 (-0.68 to -0.03)	0.026

SD = standard deviation; AAPC = average annual percentage change, CI = confidence interval

Supplementary Table 6: Prevalence of individual antihypertensive medication use in those diagnosed with hypertension, 2003-2021.

Measure	Year														Join-points	% change per year (+ means increase, - means decrease AAPC (95% CI)	*p*-value for trend
	**2003**	**2006**	**2009**	**2010**	**2011**	**2012**	**2013**	**2014**	**2015**	**2016**	**2017**	**2018**	**2019**	**2021**			
Number of individuals with diagnosed hypertension (n/%)	25.5	25.4	24.6	25.5	27.8	23.9	24.7	23.8	24.1	23.6	26.2	24.5	23.5	24.4			
Prevalence of ACE inhibitor use in diagnosed hypertensives (n/%)	26.7	34.5	42.1	46.1	43.9	47.7	50.0	48.5	49.6	50.2	43.7	51.5	50.7	48.3	2010	+3.60 (2.80 to 4.41)	<0.001
Prevalence of beta blocker use in diagnosed hypertensives (n/%)	24.2	23.3	21.6	20.6	18.8	21.9	22.7	19.9	21.7	20.6	20.4	23.3	21.8	21.7	2011	-0.46 (-1.90 to 0.54)	0.361
Prevalence of calcium channel blocker use in diagnosed hypertensives (n/%)	81.0	77.3	74.9	72.3	74.7	71.9	69.0	68.0	69.8	65.7	68.9	67.2	65.5	67.3	None	-1.27 (-1.57 to -1.02)	<0.001
Prevalence of diuretic use in diagnosed hypertensives	31.0	31.7	26.9	29.6	24.4	26.2	27.5	26.8	21.3	21.6	18.6	22.2	19.4	15.9	None	-3.02 (-4.54 to -1.60)	<0.001

(n/%)																	

SD = standard deviation; AAPC = average annual percentage change, CI = confidence interval.

Supplementary Table 7: Antihypertensive use by age (<55 or >55) and drug category, 2003-2021.

Measure		Year														Join-points	% change per year (+ means increase, - means decrease AAPC (95% CI)	*p*-value for trend
		**2003**	**2006**	**2009**	**2010**	**2011**	**2012**	**2013**	**2014**	**2015**	**2016**	**2017**	**2018**	**2019**	**2021**			
Number of individuals with diagnosed hypertension (n/%)	<55	791 (13.7)	772 (14.2)	205 (11.3)	350 (11.9)	438 (14.8)	280 (10.0)	347 (10.8)	286 (9.8)	282 (10.1)	274 (10.5)	335 (12.7)	230 (9.6)	238 (9.6)	106 (12.2)	None	-2.00% (-3.30 to -0.99%)	<0.001
	≥55	1505 (46.7)	1406 (45.2)	487 (48.5)	824 (49.9)	850 (50.9)	765 (48.8)	898 (48.6)	801 (48.4)	782 (48.3)	708 (45.5)	779 (47.8)	725 (48.1)	717 (45.5)	250 (42.7)	2011	-0.08% (-0.95 to 0.31%)	0.510
Prevalence of ACE inhibitor use in diagnosed hypertensives (n/%)	<55	90 (11.5)	133 (17.3)	58 (28.7)	109 (31.4)	117 (26.7)	85 (30.5)	122 (35.2)	87 (30.6)	85 (30.3)	90 (32.8)	91 (27.2)	83 (36.1)	85 (35.8)	34 (32.1)	2009	+6.49% (4.98 to 7.83)	<0.001
	≥55	521 (34.7)	618 (43.9)	232 (47.8)	431 (52.4)	448 (52.7)	412 (54.0)	500 (55.7)	439 (54.8)	442 (56.5)	402 (56.9)	396 (50.9)	409 (56.4)	398 (55.6)	138 (55.2)	2011	+5.36% (4.04 to 7.81%)	<0.001
Prevalence of beta blocker use in diagnosed hypertensives (n/%)	<55	94 (11.9)	89 (11.6)	20 (10.1)	26 (7.6)	37 (8.6)	29 (10.4)	48 (13.8)	22 (7.9)	27 (9.7)	34 (12.5)	33 (10.0)	21 (9.3)	20 (8.6)	9 (9.2)	None	-1.14% (-3.07 to 0.32%)	0.106
	≥55	462 (30.7)	418 (29.8)	128 (26.4)	214 (26.1)	204 (24.0)	199 (26.1)	235 (26.2)	194 (24.2)	203 (26.1)	167 (23.7)	194 (24.9)	201 (27.8)	187 (26.2)	67 (27.1)	2011	-0.93% (-1.62 to -0.16)	0.018
Prevalence of calcium channel blocker use in diagnosed hypertensives (n/%)	<55	737 (93.2)	713 (92.4)	180 (87.9)	302 (86.3)	380 (86.8)	242 (86.3)	283 (81.7)	238 (83.3)	245 (86.9)	221 (80.5)	283 (84.6)	193 (84.0)	193 (81.0)	80 (74.8)	None	-0.90% (-1.28 to -0.68%)	<0.001
	≥55	1122 (74.6)	970 (69.0)	338 (69.4)	547 (66.4)	581 (68.4)	509 (66.6)	575 (64.1)	501 (62.6)	497 (63.7)	424 (60.0)	484 (62.2)	448 (61.9)	432 (60.3)	160 (64.1)	None	-1.21% (-1.59 to 0.86%)	<0.001
Prevalence of diuretic use in diagnosed hypertensives (n/%)	<55	81 (10.3)	77 (10.0)	16 (8.3)	35 (10.3)	41 (9.5)	22 (7.9)	40 (11.7)	28 (10.1)	16 (5.9)	18 (6.8)	17 (5.3)	19 (8.2)	8 (3.6)	8 (8.1)	None	-2.52% (-5.81 to 0.01%)	0.051
	≥55	631 (41.9)	613 (43.6)	169 (34.8)	311 (37.8)	272 (32.0)	252 (33.0)	301 (33.6)	262 (32.7)	209 (26.8)	193 (27.3)	190 (24.4)	192 (26.6)	176 (24.6)	48 (19.3)	None	-3.60% (-4.86 to 2.59%)	<0.001

Supplementary Figure 1: Hypertension prevalence in England according to ’period’ and ’current’ definitions, 1994-2021.

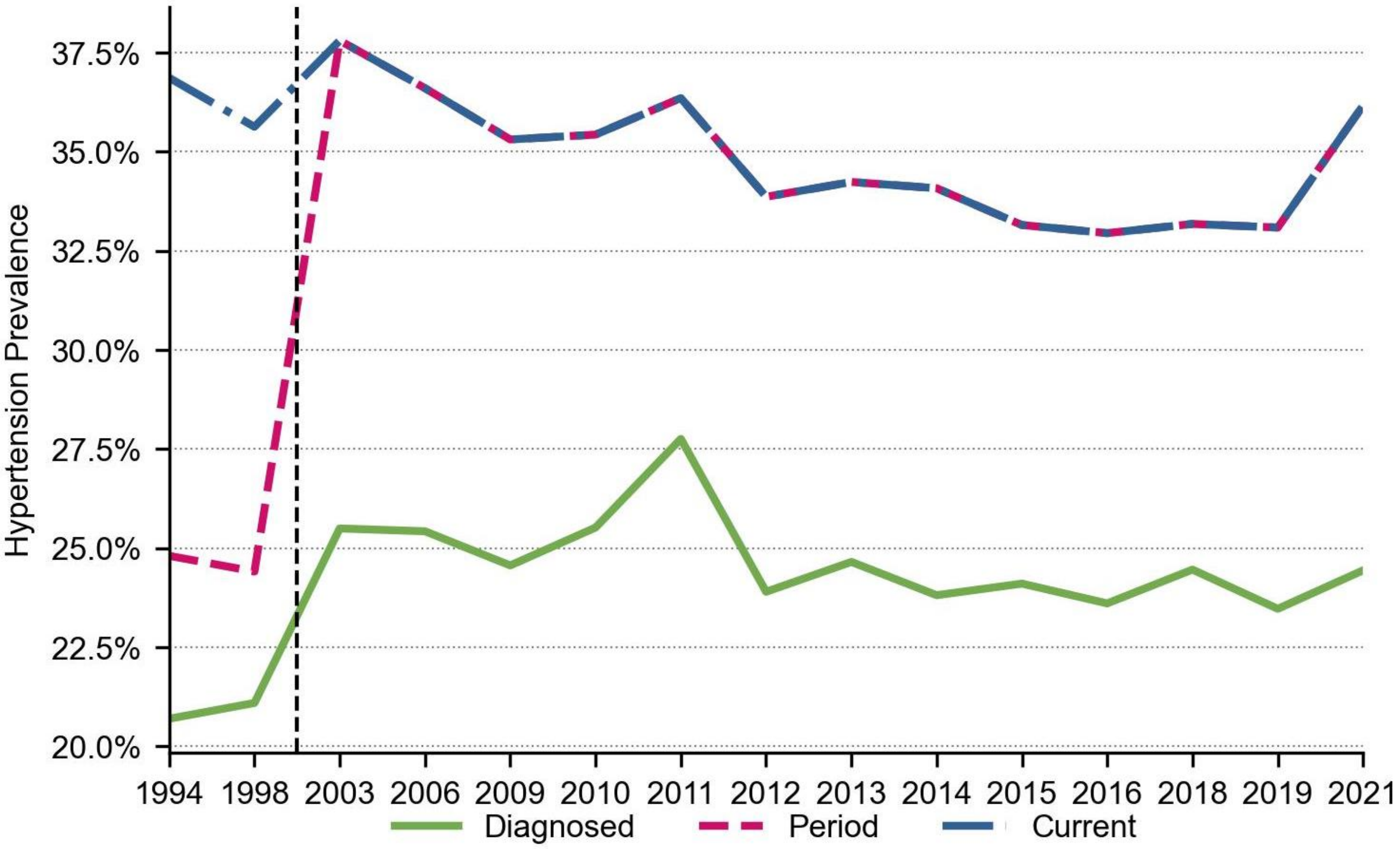

Supplementary Figure 2: Hypertension control in England, according to 'period' definitions of diagnosis and control, 1994-2021.

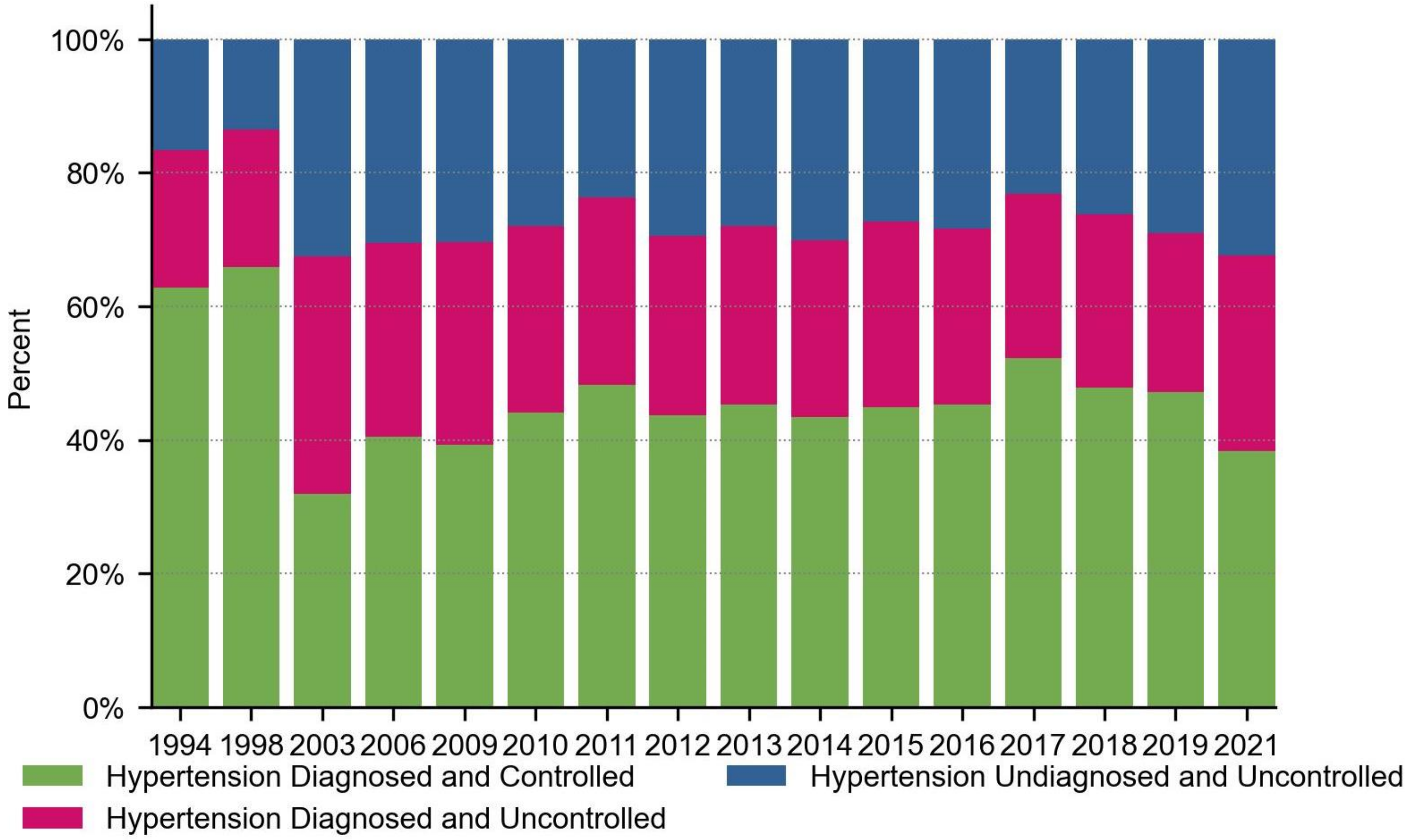

Supplementary Figure 3: Hypertension control in England, according to current definitions of diagnosis and control, 1994-2021.

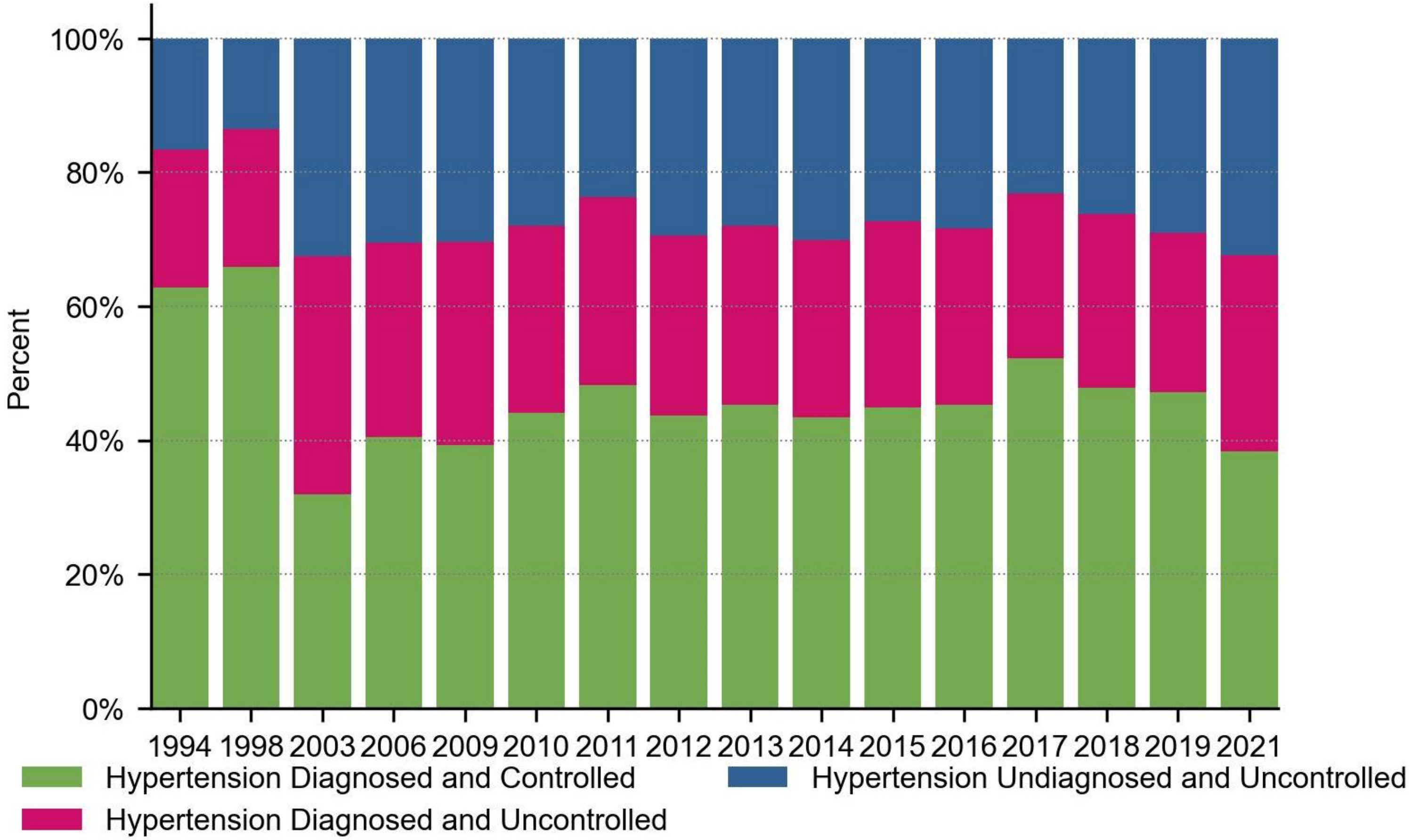

Supplementary Figure 4: Mean SBP (mmHg) in England, 1994-2021.

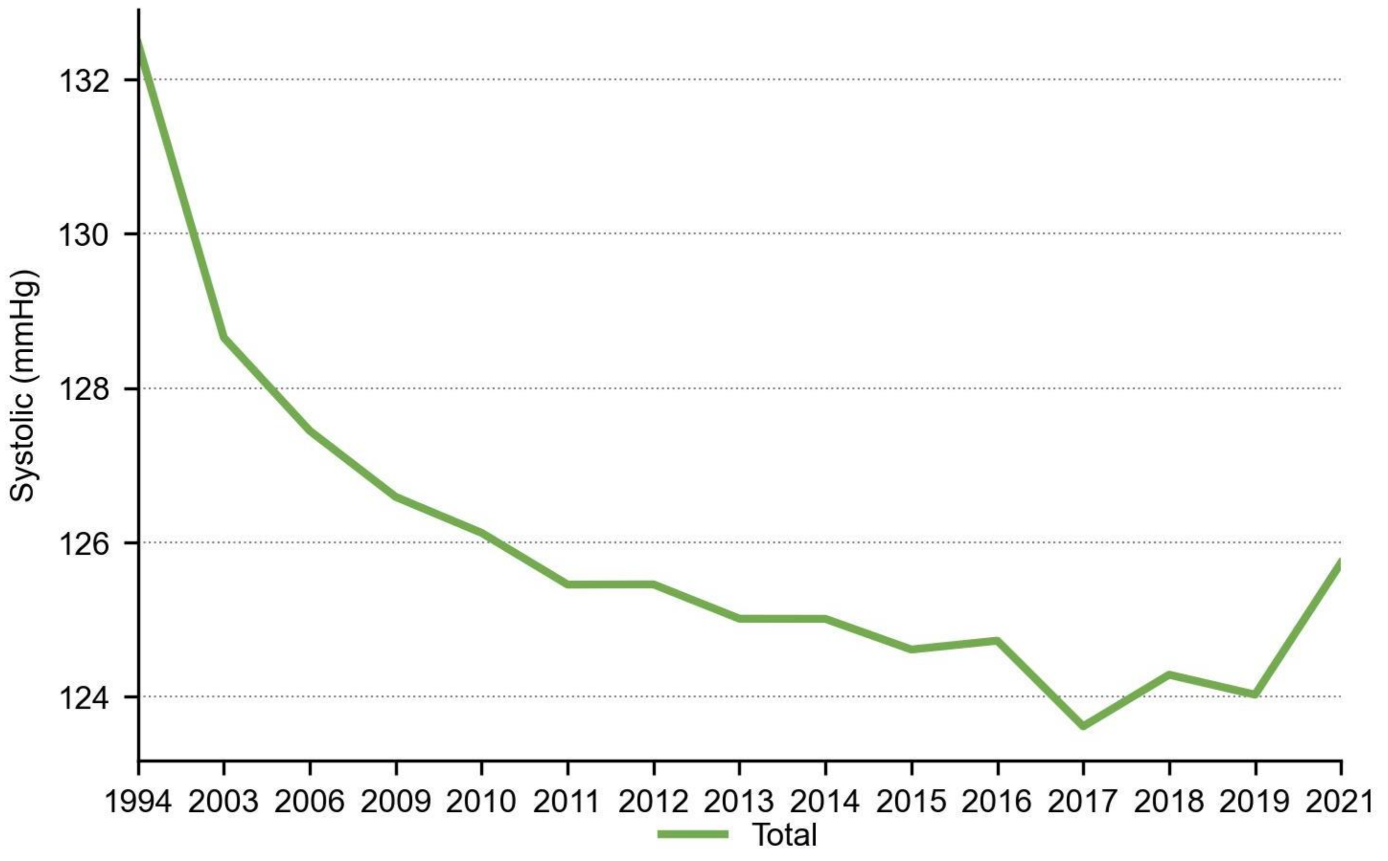

Supplementary Figure 5: Mean DBP (mmHg) in England, 1994-2021.

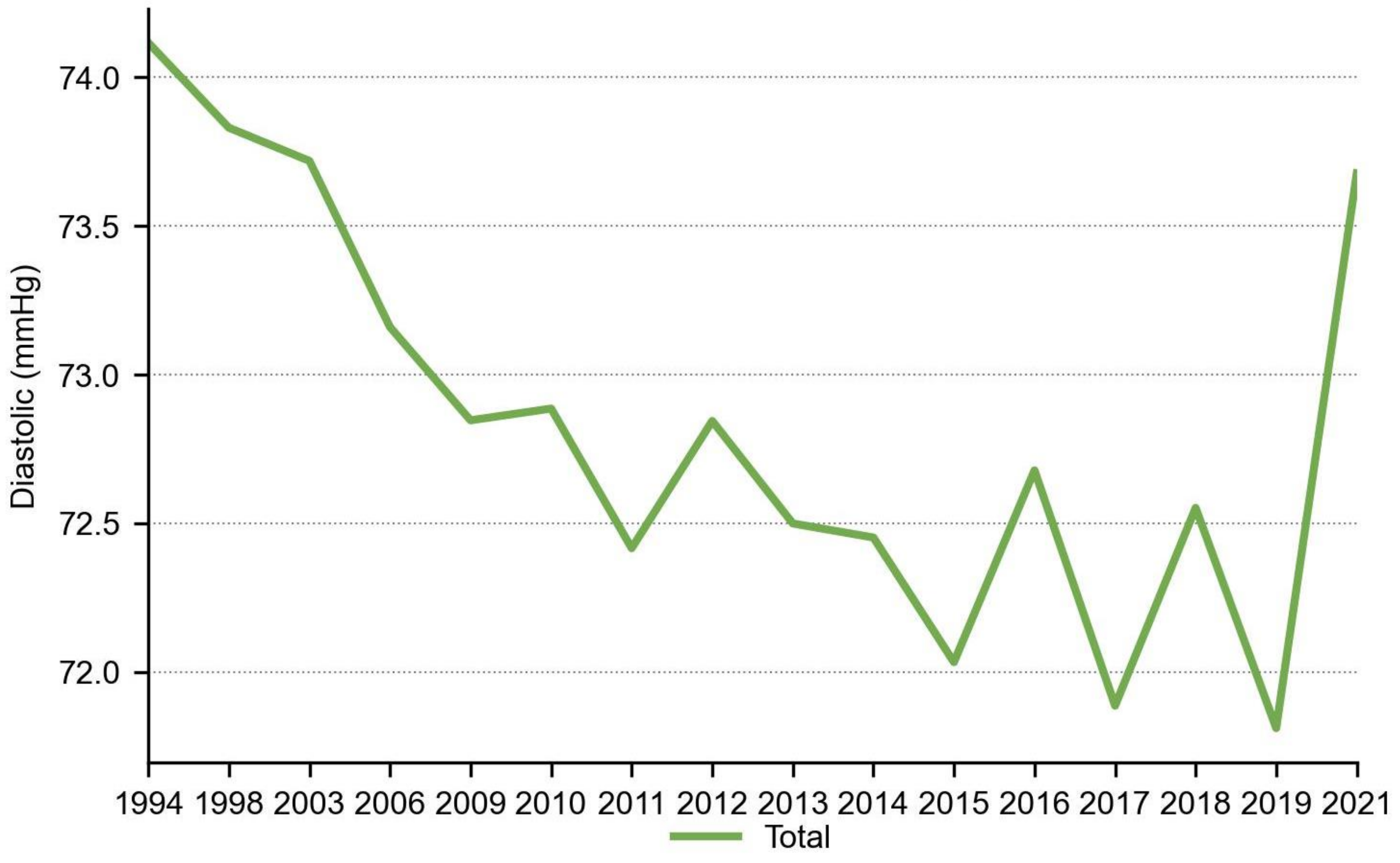

Supplementary Figure 6: Mean SBP (mmHg) in England amongst those with and without hypertension, according to period definition, 2003-2021.

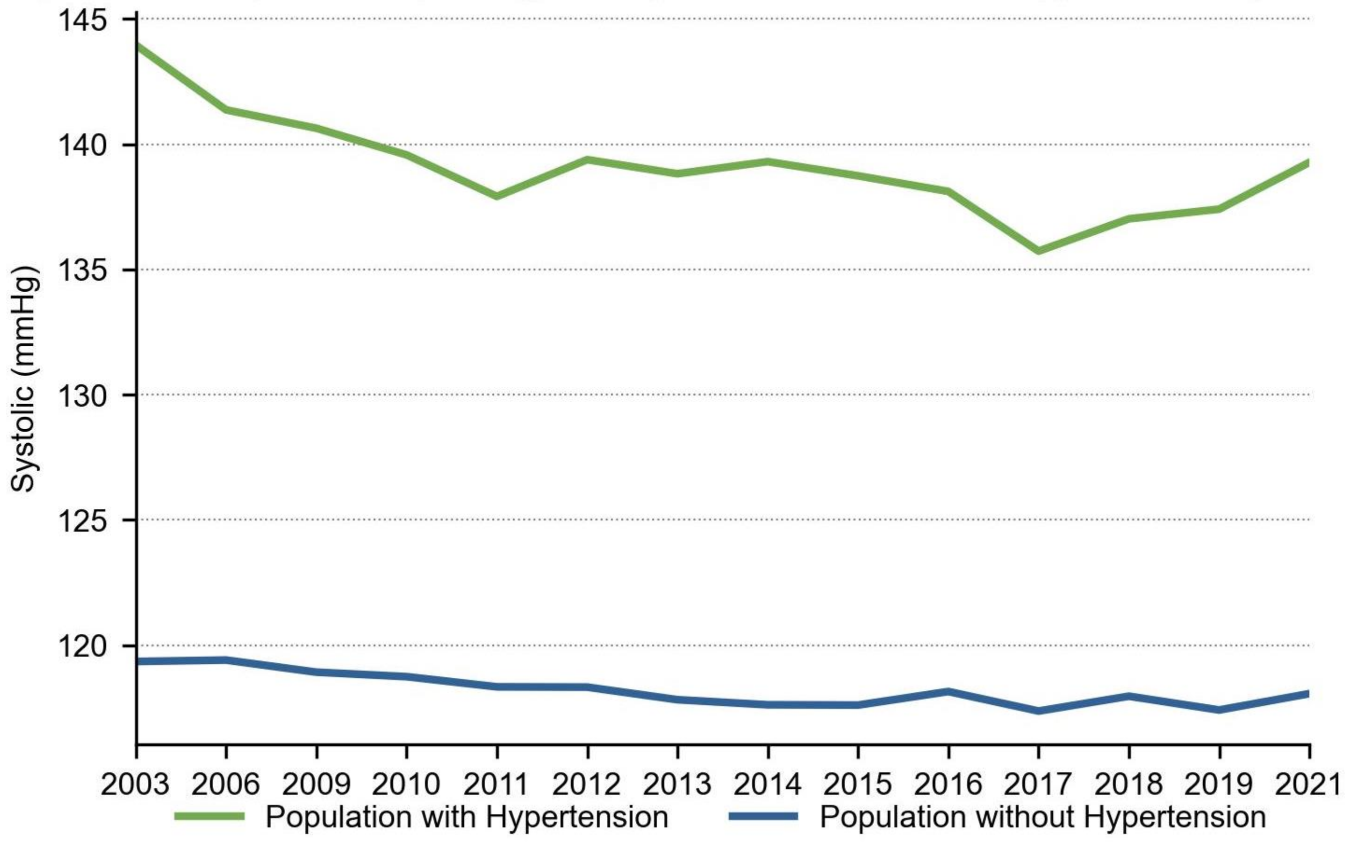

Supplementary Figure 7: Mean DBP (mmHg) in England amongst those with and without hypertension according to period definition, 2003-2021.

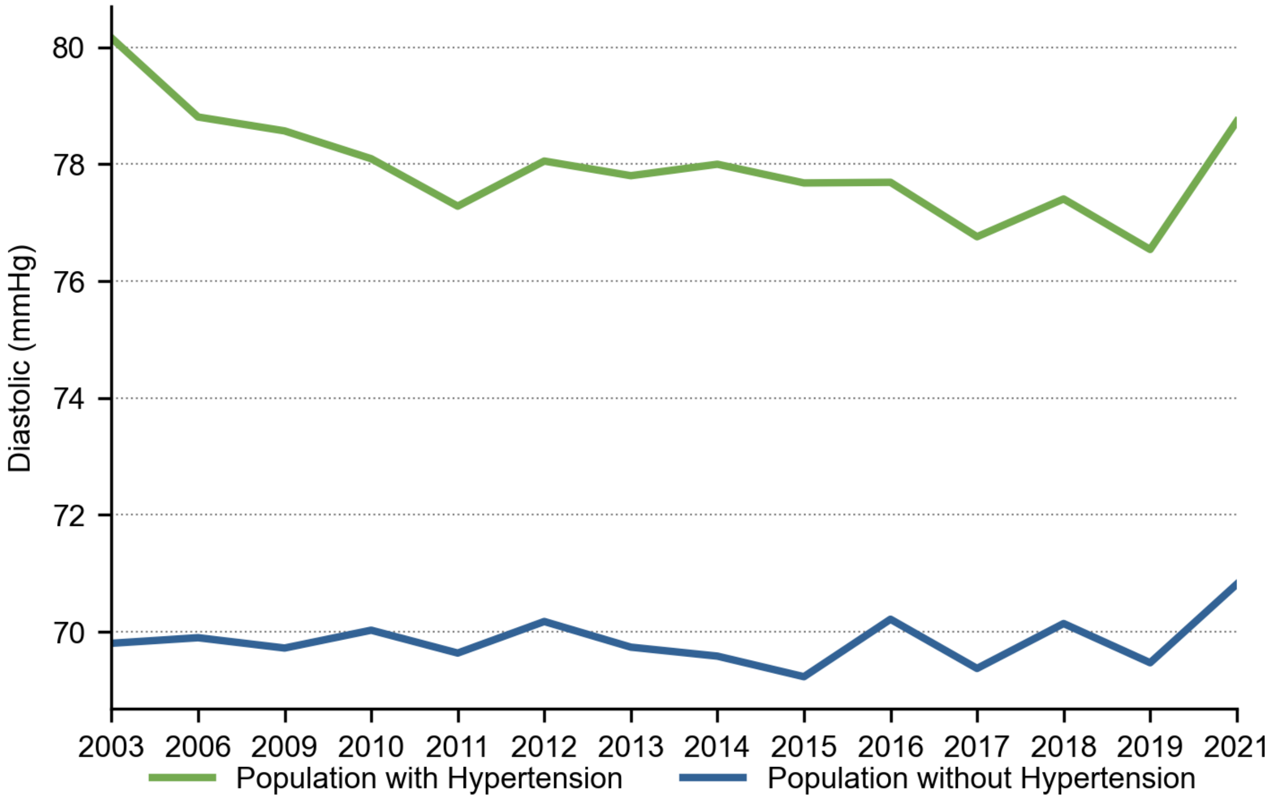

Supplementary Figure 8. Prevalence of antihypertensive use among diagnosed hypertensives.

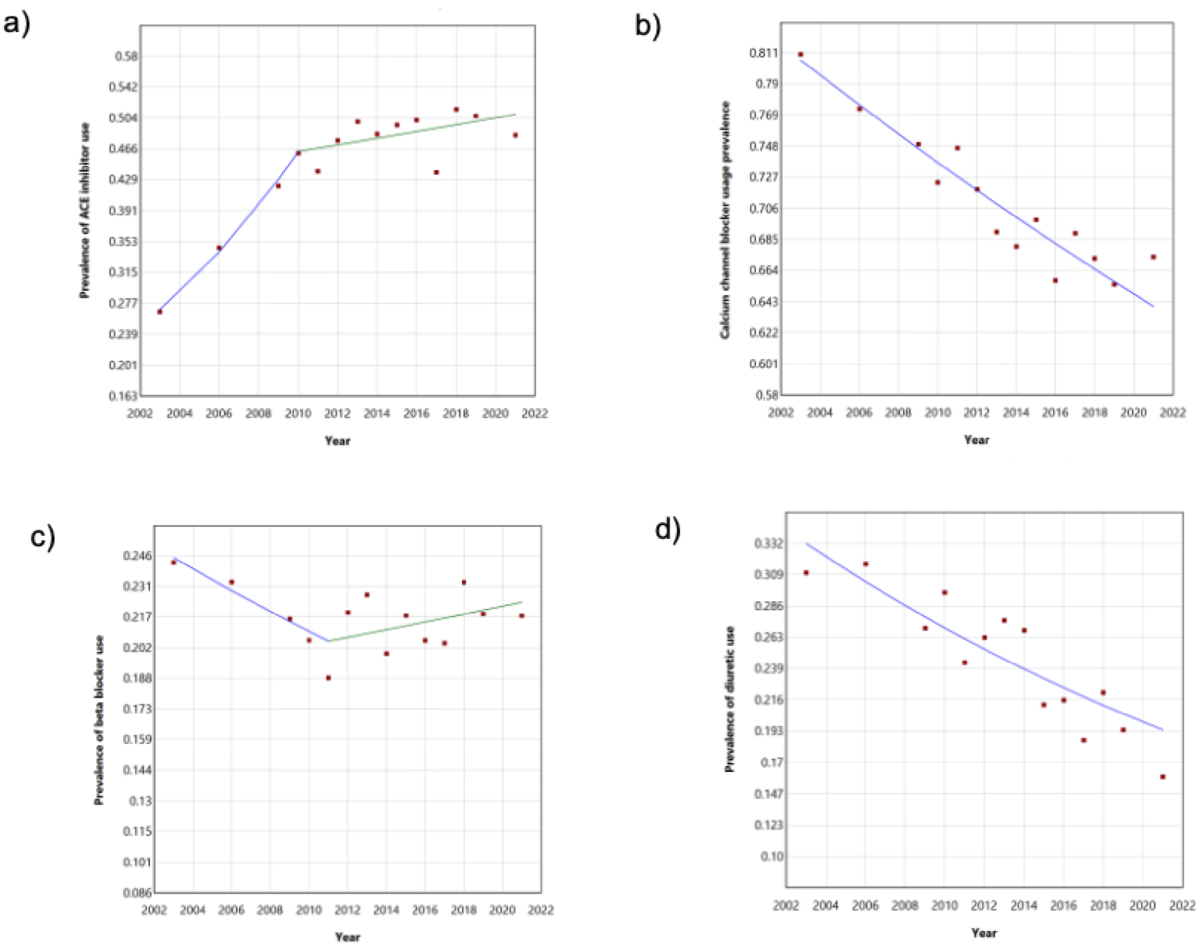

a) Prevalence of ACE inhibitor use among diagnosed hypertensives*, *b)* Prevalence of CCB use among diagnosed hypertensives, *c)* Prevalence of beta-blocker use among diagnosed hypertensives**, *d)* Prevalence of diuretic use among diagnosed hypertensives. ACE = angiotensin-converting enzyme; CCB = calcium-channel blocker.

*2003-2010 APC +8.02% (5.85 to 11.49%, $p<0.001$)). 2010-2021 APC +0.89% (-0.72 to 1.77%, $p=0.234$). **2003-2011 APC -2.13% (-4.40 to -0.36%, $p=0.018$), 2011-2021 APC +0.89% (-7.25 to 7.65%, $p=0.192$)

Supplementary Figure 9. Hypertension prevalence and overall premature cardiovascular mortality in England, 2003-2022.

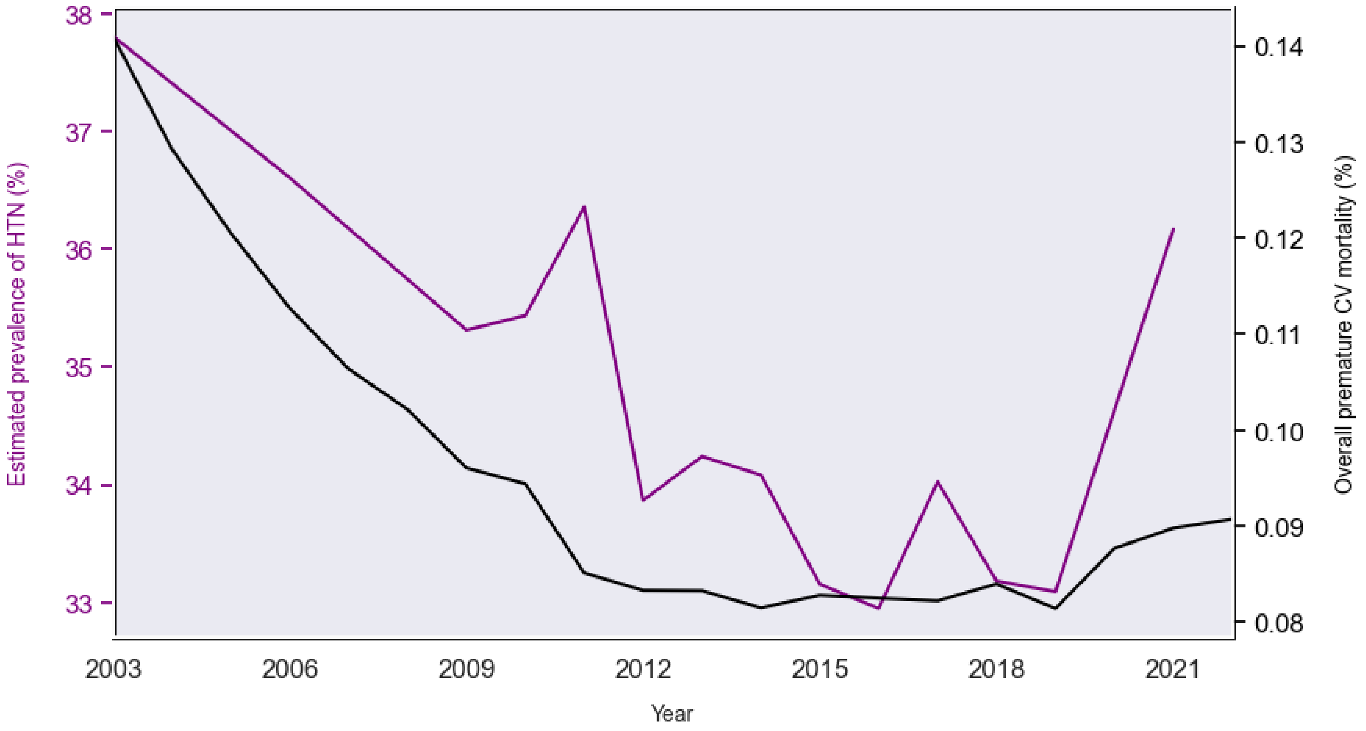

Supplement: Supplementary file 1 [file bmjmed-4-1-s001.pdf]
