# Supplementary material for: Trends in hypertension prevalence, control, and antihypertensive use in England from 2003 to 2021: insights from annual, nationwide Health Surveys for England
Source: BMJ Med. 2025 Nov 27;4(1):e001556. doi: 10.1136/bmjmed-2025-001556 (PMC12666190; doi:10.1136/bmjmed-2025-001556)
Supplement: Supplementary file 3 [file bmjmed-2025-001556.reviewer_comments.pdf]

## PEER REVIEW HISTORY

BMJ Medicine publishes all reviews undertaken for accepted manuscripts. Reviewers are asked to complete a checklist review form and are provided with free text boxes to elaborate on their assessment. These free text comments are reproduced below.

## ARTICLE DETAILS

| | |
|---|---|
| **TITLE (PROVISIONAL)** | Trends in hypertension prevalence, control and antihypertensive use in England over the last 2 decades: insights from annual, nationwide Health Surveys for England from 2003 to 2021 |
| **AUTHORS** | Gupta, Ajay; Graham, Catherine; Steckelmacher, James; Prashar, Jai; Ahmed, Ayesha; Capel, Maxim; Poulter, Neil; Sever, Peter |

## VERSION 1 - REVIEW

| | |
|---|---|
| **REVIEWER NAME** | Faconti, Luca |
| **REVIEWER AFFILIATION** | King's College London School of Medicine |
| **REVIEWER CONFLICT OF INTEREST** | None |
| **AI DISCLOSURE** | No |
| **DATE REVIEW RETURNED** | 15-Apr-2025 |

| | |
|---|---|
| **GENERAL COMMENTS** | I read with great interest the manuscript titled "Trends in Hypertension Prevalence, Control and Antihypertensive Use in England Over the Last Two Decades: Insights from Annual, Nationwide Health Surveys for England from 2003 to 2021." The authors aimed to examine decadal trends in hypertension diagnosis, control, and antihypertensive medication use in England, drawing on data from the Health Survey for England between 2003 and 2021. The analysis included a total of 67,242 individuals aged 16 and over. The key findings indicate a marked improvement in hypertension prevalence and control during the 2000s, followed by a plateau from 2011 onward. Similarly, the proportion of undiagnosed hypertension in the community declined in the early 2000s and 2010s but has since returned to levels observed two decades ago. The topic is highly relevant, and the study is methodologically sound. My main comment is with the interpretation of the observed worsening of blood pressure control in 2021 which was still importantly influenced by government restrictions and social distancing. Of note, some data from Integrated Care Boards (ICBs) in England reflect a similar trend in blood pressure control from 2019 to 2021, followed by improvement after 2022. This suggests that the observed deterioration could reflect the short-term impact of the COVID-19 pandemic, particularly the reduction in face-to-face healthcare visits. Whether this negative effect on blood pressure control will persist remains to be seen at national level and should be reassessed once data beyond 2022 become available. I would therefore suggest to review the discussion based on this aspect.<br>It could also be interesting for the readers to stratify the analysis of blood pressure trends by ethnicity and age group, as this could offer additional insights into population-level disparities.<br>I found certain aspects of the discussion to be somewhat speculative. For instance, on page 11, line 25, the authors refer to the “prohibitive cost of medications” without providing socioeconomic data to support this view. Similarly, on page 13, line |

| | |
|---|---|
| | 57, the suggestion that more patients are achieving blood pressure targets with monotherapy lacks supporting evidence within the manuscript. Additionally, the interpretation of the analysis of antihypertensive drug classes might need some insight by age and ethnic group, given that current NICE guidelines recommend ACE inhibitors or ARBs as first-line treatment for < 55 years non African origin or subjects with diabetes. |

| | |
|---|---|
| **REVIEWER NAME** | Fujiwara, Takeshi |
| **REVIEWER AFFILIATION** | University of Oxford, Nuffield Department of Primary Care Health Sciences |
| **REVIEWER CONFLICT OF INTEREST** | None |
| **AI DISCLOSURE** | No |
| **DATE REVIEW RETURNED** | 17-Apr-2025 |

| | |
|---|---|
| **GENERAL COMMENTS** | Summary<br>The aim of this nationwide observational study in England was to investigate the prevalence of hypertension, blood pressure (BP) levels, and changes of the number of antihypertensive medication use between 2003 and 2021. This study showed that the prevalence of hypertension decreased, and BP levels decreased until 2011, and then a plateau, and a possible worsening after the pandemic.<br><br>Comments<br>・Compared to the previous findings, especially the paper the authors cited (Lancet. 2014; 31;383:1912–9), the authors should describe the novelty of this study.<br>・The reviewer believes that the contents of COVID-19 pandemic was abrupt in the purpose of the introduction section, and the authors should have clearly explained how the pandemic has affected health care and why it was necessary to examine the association with BP control before the description of the purpose of this study.<br>・This reviewer also considers that it is necessary to explain in the introduction section why it is necessary to investigate changes in the categories of antihypertensive drugs over time.<br>・In the discussion section, various factors that affect the prevalence of hypertension and BP control were mentioned. However, this reviewer believes that investigating these factors in detail is an important point that contributes to decreasing the prevalence of hypertension and increasing the BP control rate. This study seems more like a report than a scientific paper.<br>・Based on the results of this study, the authors should specifically state what important measures society should take to increase the rate of BP control. |

| | |
|---|---|
| **REVIEWER NAME** | Davies, Neil |
| **REVIEWER AFFILIATION** | UCL, Division of Psychiatry and Department of Statistical Science |
| **REVIEWER CONFLICT OF INTEREST** | None |
| **AI DISCLOSURE** | No |
| **DATE REVIEW RETURNED** | 16-May-2025 |

| | |
|---|---|
| **GENERAL COMMENTS** | This is an excellent and important study, that highlights potentially important changes in blood pressure, hypertension and anti-hypertensive treatment over time. The key limitation of the study is the 2021 results were generated via different methods, with a different sample size and in took place during the COVID pandemic. As a result, I agree with the authors cautious interpretation of these results and conclusions. We need more data from future years to see if this increase in rates is an artifact of COVID, the measurement methodology or a true change in the prevalence of undiagnosed hypertension. Either way this remains an important paper.<br>Below, I provide some comments that I hope help improve the paper:<br><br>Major<br>1. Why is the sample size so much smaller in 2021? Would be worth explaining the drop in sample size – why this occurred (COVID?). The results and the increasing trend since COVID are a based on a small sample, and we need more data from future years to follow this. However, the stagnation in primary prevention of hypertension in the population for the years 2010-2019 is clear in the data presented.<br><br>Minor<br>1. P3L27 – prevalence of *diagnosed* hypertension or measured hypertension? Could you clarify that your objective is estimate true rates of hypertension in the population, not just diagnosed? Maybe also clarify that your definition of hypertension refers to untreated or any hypertension? (if this is correct…)<br>2. P3L55 – why “possible” here – did you detect a worsening or not? This is probably ok as phrased, and reflects the nuanced take in your discussion.<br>3. P4l25 – This sentence is too long and possibly lacks a “cost saving” element, and your point gets lost (hypertension treatments are cost-effective). I suggest splitting it.<br>4. P4L33 “wider *UK* economy.<br>5. P4L38 – this para goes back to a global take – I would suggest dropping and amalgamating with the first.<br>6. P5L10 – maybe add “with directly measured blood pressure” – this is one of the key contributions of your study and allows you to estimate rates of underdiagnosis.<br>7. P5L22 – maybe change to just the change in blood pressure trends after the pandemic as ascribing cause will be tricky and not really necessary for your purposes.<br>8. P6L42 – maybe convert this into a number list or bullet points.<br>9. P7L26 – are new people survey in each survey, or is this a longitudinal panel?<br>10. P8L12 – Consider making a comment about multiple testing here – do you have a primary hypothesis? To be honest, correcting for multiple testing is rarely that pivotal, but you might want to explain your rationale for 0.05 here. Alternatively, could just say you’ll interpret the CIs rather than focus on the P-values.<br>11. P8L46 – this is a surprisingly large fraction without valid BP measurements, why is this?<br>12. Figures and tables – you don’t present SEs or CIs, is this because they’re very precisely estimated? Or another reason? Maybe justify this omission in the notes.<br>13. P9L12 – you can drop discussion of statistically significant here, just report the trend you have detected, then you can conclude that there was little evidence of a increase in 2017-2021. |

| | |
|---|---|
| | 14. P9l32 – so there was evidence that hypertension decreased 2003-2021, but there was little evidence it increased 2018-2021<br>15. P10L7 – “apparent surge” – just test for difference and tell your reader whether there’s evidence of an increase or not.<br>16. P10L12 – the table has N/% for number without BP control. But I only see %.<br>17. P5L51 Was the study pre-registered? Add the details here of your approval – committee that approved it and the number of your study (if there is one).<br>18. Can you add the code used to clean and analyse your data to a repo like GitHub?<br>19. Did anyone perform a code review?<br>20. Can you add the details of the ethical approval and the committee that approved the analysis? |

## VERSION 1 – AUTHOR RESPONSE

**Reviewer: 1**
**Luca Faconti, King's College London School of Medicine**
**Comments to the Author**
I read with great interest the manuscript titled "Trends in Hypertension Prevalence, Control and Antihypertensive Use in England Over the Last Two Decades: Insights from Annual, Nationwide Health Surveys for England from 2003 to 2021." The authors aimed to examine decadal trends in hypertension diagnosis, control, and antihypertensive medication use in England, drawing on data from the Health Survey for England between 2003 and 2021. The analysis included a total of 67,242 individuals aged 16 and over. The key findings indicate a marked improvement in hypertension prevalence and control during the 2000s, followed by a plateau from 2011 onward. Similarly, the proportion of undiagnosed hypertension in the community declined in the early 2000s and 2010s but has since returned to levels observed two decades ago.
The topic is highly relevant, and the study is methodologically sound.

**We thank the reviewer for their comments and interest in the manuscript.**

My main comment is with the interpretation of the observed worsening of blood pressure control in 2021 which was still importantly influenced by government restrictions and social distancing. Of note, some data from Integrated Care Boards (ICBs) in England reflect a similar trend in blood pressure control from 2019 to 2021, followed by improvement after 2022. This suggests that the observed deterioration could reflect the short-term impact of the COVID-19 pandemic, particularly the reduction in face-to-face healthcare visits. Whether this negative effect on blood pressure control will persist remains to be seen at national level and should be reassessed once data beyond 2022 become available. I would therefore suggest to review the discussion based on this aspect.

**Thank you for your insightful comment; we agree this is a limitation. At the time of the 2021 survey, government restrictions and social distancing measures had not been fully lifted. We have added further clarification on this in discussion of the limitations of the study in the manuscript. We have explained in the discussion the importance of analysing the 2022 datasets and beyond once they are available (they are not available currently and are expected to be released by the end of 2025/early 2026).**

**While it is difficult to predict precisely what the 2022 data will reveal, we would like to highlight a key observation from the existing trend: following notable improvements between 2003 and 2011, a plateau appears to have emerged thereafter. The deterioration observed post-2019 may therefore represent an exacerbation of this underlying stagnation. Whether this decline is transient or marks a sustained shift remains to be seen.**

**We share the reviewer's hope that the forthcoming HSE data may corroborate the more encouraging trends noted in the ICB data from England, as referenced in your comment, and potentially indicate a degree of post-pandemic recovery. It will be particularly informative to examine whether these indicators return to pre-2019 levels or whether a more persistent impact of the pandemic is evident.**

**Of further interest, recent US data (Fryar et al., 2024; PMID: 40085792) provide a valuable point of comparison. This study, using a different methodology from ours, found no significant change in hypertension prevalence, awareness, treatment, or control when comparing the pre-pandemic period (2017–March 2020) with the post-pandemic period (August 2021–August 2023), excluding the period most affected by pandemic-related restrictions. It will be interesting to see whether the forthcoming HSE data for 2022 and 2023 align with these US findings.**

**In response to the reviewer's suggestion, we have now incorporated this element of uncertainty and reflection into the *Limitations* to better acknowledge the evolving and complex nature of these trends.**

It could also be interesting for the readers to stratify the analysis of blood pressure trends by ethnicity and age group, as this could offer additional insights into population-level disparities.

**We thank the reviewer for their comment and suggestion and agree that this analysis would be informative. Whilst we have grouped ethnicities in table 1 of baseline characteristics, ethnicity was defined differently across surveys and was not consistent across the study period. Additionally, the groups for some ethnicities are very small so it is difficult to draw meaningful conclusions from these. We agree with the reviewer that it would be helpful to have analysis by age group (<55 years / > 55 years) to match with NICE guidelines on treatment recommendations, so this is now included in the manuscript. See supplementary table 7, which shows that in England, amongst those < 55 years of age, the use of CCBs has reduced since 2006 with associated increase in the use of ACE inhibitors in the same population.**

I found certain aspects of the discussion to be somewhat speculative. For instance, on page 11, line 25, the authors refer to the "prohibitive cost of medications" without providing socioeconomic data to support this view. Similarly, on page 13, line 57, the suggestion that more patients are achieving blood pressure targets with monotherapy lacks supporting evidence within the manuscript.

**We thank the reviewer for their comments on these aspects and have removed them from the discussion.**

Additionally, the interpretation of the analysis of antihypertensive drug classes might need some insight by age and ethnic group, given that current NICE guidelines recommend ACE inhibitors or ARBs as first-line treatment for < 55 years non African origin or subjects with diabetes.

**Thanks, in response, we have now performed an analysis by age and included this in *Supplementary Table* 7. While an analysis by ethnic group would certainly be interesting in view of the NICE guidelines, we found that the subset of treated hypertensives in the HSE sample was rarely sufficiently powered to enable this. In addition, the definition and classification of ethnic groups have varied over the course of this evaluation, making direct comparisons of trends between different annual surveys challenging.**

**Reviewer: 2**
**Dr. Takeshi Fujiwara, University of Oxford**
**Comments to the Author**
Summary
The aim of this nationwide observational study in England was to investigate the prevalence of hypertension, blood pressure (BP) levels, and changes of the number of antihypertensive medication use between 2003 and 2021. This study showed that the prevalence of hypertension decreased, and BP levels decreased until 2011, and then a plateau, and a possible worsening after the pandemic.

**We thank the reviewer for their summary of the manuscript and their comments below.**

Comments
・Compared to the previous findings, especially the paper the authors cited (Lancet. 2014; 31;383:1912–9), the authors should describe the novelty of this study.

**We agree that it is important that this study demonstrates novelty compared to the previous study (Falaschetti et al. Lancet. 2014: 31;383:1912–9). The study by Falaschetti et al. painted a relatively promising picture from the 1990s and 2000s and projected that if improvements continued at that pace 80% of patients would have satisfactory BP control by 2022. Our study clearly refute that prediction, demonstrating the extent of this overestimation. Furthermore, we have clearly outlined in the *Introduction* that our study aims to evaluate if these improvements were sustained beyond 2011 and situate this study in the context of the rising trend of premature cardiovascular mortality rates in recent years, partially offsetting the previously accrued gains..**

**The study also provides some initial data on BP trends around the COVID pandemic though we agree that further data from 2022 and beyond once available will be important to assess this further. The further novelty of this study is that we assess treatment trends within the context of different antihypertensive medication classes for which guidance changed in the mid to late 2000s following the ASCOT study (in 2006 guidance changed on anti-HTN meds, from 'ABCD' to 'ACD' and 2011 BIHS and NICE thrust to use guidance based medications.)**

**In response to your comments, we have added some clarifications on these points in the manuscript, particularly in line with the BIHS and NICE guidance around age cut-off of >55 years, and those below.**

**We think that these constitute substantially novel additions to the epidemiological understanding of hypertension in England since 2011, and the study by Falaschetti et al.**

・The reviewer believes that the contents of COVID-19 pandemic was abrupt in the purpose of the introduction section, and the authors should have clearly explained how the pandemic has affected health care and why it was necessary to examine the association with BP control before the description of the purpose of this study.

**We thank the reviewer for their comment and agree that this section could be improved. Most obviously during the COVID-19 pandemic, acute services were put under unprecedented strain during the initial waves, but the effect on prevention and management of chronic conditions is often not truly understood until after the fact.**

**The majority of our data in this study comes from the pre-pandemic surveys with preliminary post-pandemic data in a smaller survey from 2021. A further study with data from 2022 and 2023 once available will be a better assessment of the effect of the COVID-19 pandemic on BP**

**control. We have added some clarifications on these points the manuscript (see paragraph 4 of the introduction.)**

・This reviewer also considers that it is necessary to explain in the introduction section why it is necessary to investigate changes in the categories of antihypertensive drugs over time.
・In the discussion section, various factors that affect the prevalence of hypertension and BP control were mentioned. However, this reviewer believes that investigating these factors in detail is an important point that contributes to decreasing the prevalence of hypertension and increasing the BP control rate. This study seems more like a report than a scientific paper.
・Based on the results of this study, the authors should specifically state what important measures society should take to increase the rate of BP control.

**We thank the reviewer for their comments. We hope that the manuscript provokes further conversations about how the various factors, including changes in prescribing practices, impact hypertension prevalence, diagnosis and control.**

**We have added detail in the introduction (paragraph 4) on why it is necessary to investigate changes in the categories of antihypertensive drugs over time – specifically as during the period studies there were significant updates to the treatment algorithms after the publication of seminal studies such as ASCOT. We would draw attention to our explorations of correlations with e.g. salt consumption in the discussion as further evidence for the changes observed.**

**We feel that a key take-home message of our analysis is that ground has been lost in the 2010s (in the 'austerity' era) and into the COVID-19 pandemic, since significant gains were made in the 1990s and 2000s. We suggest how societal changes may have contributed to this and indeed discuss some of the social determinants but we also recognise that this is a complex issue to solve.**

**Gains made in the 1990s and 2000s required a multi-agency approach with stakeholders throughout society; we touch on the importance of this in the discussion, alongside evaluation of the factors which may have led to improvements in BP across society over this time. Detailed policy suggestions are beyond the scope of our study, but we expect these findings to contribute to the evidence base that prompts action and discussions around future policy direction and priorities.**

**Reviewer: 3**
**Prof. Neil Davies, UCL**
**Comments to the Author**
bmjmed-2025-001556 Trends in hypertension prevalence, control and antihypertensive use in England over the last 2 decades: insights from annual, nationwide Health Surveys for England from 2003 to 2021

This is an excellent and important study, that highlights potentially important changes in blood pressure, hypertension and anti-hypertensive treatment over time. The key limitation of the study is the 2021 results were generated via different methods, with a different sample size and in took place during the COVID pandemic. As a result, I agree with the authors cautious interpretation of these results and conclusions. We need more data from future years to see if this increase in rates is an artifact of COVID, the measurement methodology or a true change in the prevalence of undiagnosed hypertension. Either way this remains an important paper.

**We thank the reviewer for their comments on the study and the time they have taken to review the manuscript.**

Below, I provide some comments that I hope help improve the paper:

Major
1. Why is the sample size so much smaller in 2021? Would be worth explaining the drop in sample size – why this occurred (COVID?). The results and the increasing trend since COVID are a based on a small sample, and we need more data from future years to follow this. However, the stagnation in primary prevention of hypertension in the population for the years 2010-2019 is clear in the data presented.

**We thank the reviewer for their comment on the limitations of the 2021 data, and echo our response to Reviewer 1. While the reason for the smaller sample is not elaborated in detail upon in the available literature, the 2021 survey took place while government pandemic restrictions were still in force, presumably limiting the ability of the study team to recruit participants and collect data. We have added further clarification on this in the *Limitations.***

**Given the plateau in improvements from 2011, the extra strain on health services during and after the pandemic perhaps represent a case of the 'straw that broke the camel's back' rather than a *de novo* effect.**

**We wholeheartedly agree that more data from the 2022 HSE and beyond will be required to draw further conclusions on the impact of the COVID-19 pandemic. It will be interesting to see if HSE data once available for 2022 and 2023 shows similar trends to comparable US datasets now available to 2023 that showed the prevalence, awareness, treatment and control of hypertension in adults has not changed significantly comparing the pre-pandemic period 2017 -March 2020 and post-pandemic period August 2021- August 2023, which excludes the bulk of government restrictions and social distancing measures in the US (Fryar et al. 2024 PMID: 40085792)**

Minor
1. P3L27 – prevalence of *diagnosed* hypertension or measured hypertension? Could you clarify that your objective is estimate true rates of hypertension in the population, not just diagnosed? Maybe also clarify that your definition of hypertension refers to untreated or any hypertension? (if this is correct…)

**We have made these clarifications in the abstract.**

2. P3L55 – why "possible" here – did you detect a worsening or not? This is probably ok as phrased, and reflects the nuanced take in your discussion.

**Yes – while the data is indicative of an effect, the trend is not significant. On balance, we felt this was the best way to reflect the nuance in this finding, and the need for further evaluation once novel data is available.**

3. P4l25 – This sentence is too long and possibly lacks a "cost saving" element, and your point gets lost (hypertension treatments are cost-effective). I suggest splitting it.

**We agree that splitting the sentence means these points are not lost.**

4. P4L33 “wider *UK* economy.

**We have checked the reference used which is the Kings Fund Report from 2022 and this cost to the wider economy is in relation to England only, not the UK. We have clarified that this relates to England.**

5. P4L38 – this para goes back to a global take – I would suggest dropping and amalgamating with the first.

**We agree that the introduction reads better when discussing the global context in full before focussing on England. We have therefore re-structured this in the re-submitted manuscript.**

6. P5L10 – maybe add “with directly measured blood pressure” – this is one of the key contributions of your study and allows you to estimate rates of underdiagnosis.

**We have added this to the manuscript.**

7. P5L22 – maybe change to just the change in blood pressure trends after the pandemic as ascribing cause will be tricky and not really necessary for your purposes.

**We have modified this aim in the manuscript.**

8. P6L42 – maybe convert this into a number list or bullet points.

**We agree that numbered categories are clearer so have made this change to the manuscript.**

9. P7L26 – are new people survey in each survey, or is this a longitudinal panel?

**New people are surveyed in each survey; this has been clarified in the first paragraph of the *Methods.***

10. P8L12 – Consider making a comment about multiple testing here – do you have a primary hypothesis? To be honest, correcting for multiple testing is rarely that pivotal, but you might want to explain your rationale for 0.05 here. Alternatively, could just say you’ll interpret the CIs rather than focus on the P-values.

**Thanks for your comments. We agree that multiple testing is rarely pivotal; our analyses were largely descriptive in respect of prevalence trends, and we have only tested for trends in that context. Furthermore, we have adjusted the text in the *Methods* to emphasise interpretation of the CIs (and the interpretation of effect size magnitude and precision) over sole reliance on p-values.**

11. P8L46 – this is a surprisingly large fraction without valid BP measurements, why is this?

**This was in part because only those patients who had x3 BP readings, after 5 mins rest, 1 min apart were included. Additionally, those who had eaten, drunk alcohol or smoked 30 mins before measurements were excluded from analyses.**

12. Figures and tables – you don't present SEs or CIs, is this because they're very precisely estimated? Or another reason? Maybe justify this omission in the notes.

**Standard errors (SEs) and 95% confidence intervals (CIs) are not shown in the main tables to improve readability; many of the estimates are precisely estimated as the reviewer implies. However, the outcome of interest is the overall trend rather than the point estimates, which is represented in the Joinpoint regression models (Figures 2-4), and all significant differences in annual trends are provided with the effect sizes with the associated 95% CI.**

13. P9L12 – you can drop discussion of statistically significant here, just report the trend you have detected, then you can conclude that there was little evidence of a increase in 2017-2021.

**We have made the suggested changes to this section in the manuscript.**

14. P9l32 – so there was evidence that hypertension decreased 2003-2021, but there was little evidence it increased 2018-2021

**We have adjusted this section in the manuscript.**

15. P10L7 – "apparent surge" – just test for difference and tell your reader whether there's evidence of an increase or not.

**Noted; there was no significant trend in our Joinpoint model for treated uncontrolled hypertension to 2021 (Figure 3), and we have clarified this in the text.**

16. P10L12 – the table has N/% for number without BP control. But I only see %.

**Thank you for highlighting this. We have adjusted the units to read '%' only (weighted percentage).**

17. P5L51 Was the study pre-registered? Add the details here of your approval – committee that approved it and the number of your study (if there is one).

**The Health Survey for England (HSE) has been conducted annually since 1991 as a government-commissioned, population-based survey designed to monitor health trends and inform national health policy. To our knowledge, the early iterations of these surveys were not registered in any international study registry. This is unsurprising, as the HSE predates the establishment of most contemporary trial or study registration platforms, including ClinicalTrials.gov (launched in 2000) and the ISRCTN registry (established in 1998).**

**Importantly, each annual HSE has received independent ethical approval (see the response to point 20 below), and full details of these approvals are provided within our manuscript. Our analysis is based on anonymised, publicly available datasets obtained from the UK Data Service. As such, our secondary data analysis did not require additional ethical approval or formal registration, in line with standard practice and institutional guidance for research involving de-identified, public domain data.**

18. Can you add the code used to clean and analyse your data to a repo like GitHub?

**We thank the reviewer for their comment. We are currently refining the code for use in other analyses, however this can of course be provided on request. We have added this statement to the manuscript.**

19. Did anyone perform a code review?

**Yes, MC wrote the original code and a code review was conducted by JP. The primary analyses were performed using Joinpoint software *(Surveillance Research Program , National Cancer Institute (2025). Joinpoint Regression Software, Version 5.4.0 - April 2025. (https://surveillance.cancer.gov/joinpoint))* rather than via original code.**

20. Can you add the details of the ethical approval and the committee that approved the analysis?

**We thank the reviewer for their comment. Ethical approval was obtained prior to each survey from the relevant Research Ethics Committees. For example, overall ethical approval for the HSE between 2016 and 2021 was obtained from the East Midlands Nottingham 2 Research Ethics Committee in 2015 (Reference no. 15/EM/0254) with the specific features of the 2021 survey reviewed and approved in October 2020. Ethical approval for the 2015 survey was obtained from the West London Research Ethics Committee (reference number 14/LO/0862). Information on REC approval for HSE prior to this can be found at https://digital.nhs.uk/data-and-information/publications/statistical/health-survey-for-england. Ethical approval was not required for our subsequent analysis and study.**

## VERSION 2 – REVIEW

| | |
|---|---|
| **REVIEWER NAME** | Faconti, Luca |
| **REVIEWER AFFILIATION** | King's College London School of Medicine |
| **REVIEWER CONFLICT OF INTEREST** | None |
| **AI DISCLOSURE** | No |
| **DATE REVIEW RETURNED** | 04-Jul-2025 |

| | |
|---|---|
| **GENERAL COMMENTS** | The authors provided detailed replied and made a few changes to the manuscript. I fully support its publication in its present form. |

| | |
|---|---|
| **REVIEWER NAME** | Fujiwara, Takeshi |
| **REVIEWER AFFILIATION** | University of Oxford, Nuffield Department of Primary Care Health Sciences |
| **REVIEWER CONFLICT OF INTEREST** | None |
| **AI DISCLOSURE** | No |
| **DATE REVIEW RETURNED** | 12-Jul-2025 |

| | |
|---|---|
| **GENERAL COMMENTS** | Issues were addressed in the present revision.<br>I have no further comments. |

| | |
|---|---|
| **REVIEWER NAME** | Davies, Neil |
| **REVIEWER AFFILIATION** | UCL, Division of Psychiatry and Department of Statistical Science |
| **REVIEWER CONFLICT OF INTEREST** | None |

| | |
|---|---|
| **AI DISCLOSURE** | No |
| **DATE REVIEW RETURNED** | 15-Jul-2025 |

| | |
|---|---|
| **GENERAL COMMENTS** | Thank you for considering my comments.<br><br>I would strongly encourage you to publish your code; this will save you time from future queries and allow others to reuse what you've done.<br><br>I would note that you performed a code review in your methods, as this is important for reproducibility. |

**VERSION 2 – AUTHOR RESPONSE**

Reviewer: 1
Luca Faconti, King's College London School of Medicine
Comments to the Author
The authors provided detailed replied and made a few changes to the manuscript. I fully support its publication in its present form.

**We thank the reviewer for their time and support in improving this manuscript**

Reviewer: 2
Dr. Takeshi Fujiwara, University of Oxford
Comments to the Author
Issues were addressed in the present revision.
I have no further comments.

**We thank the reviewer for their time and support in improving this manuscript**

Reviewer: 3
Prof. Neil Davies, UCL
Comments to the Author
Thank you for considering my comments.

I would strongly encourage you to publish your code; this will save you time from future queries and allow others to reuse what you've done.

I would note that you performed a code review in your methods, as this is important for reproducibility.

**We thank the reviewer for their time and support in improving this manuscript. We appreciate their further feedback. Specifically we have included confirmation that all statistical code was reviewed by at least two members of the team (page 7.) We agree that for the sake of transparency and reproducibility the code should be made available to others. The statistical code used for the analysis can be provided on request to the corresponding author. This is stated on page 17.**
